# The extracellular matrix glycoprotein ADAMTSL2 is increased in heart failure and inhibits TGFβ signalling in cardiac fibroblasts

**DOI:** 10.1038/s41598-021-99032-2

**Published:** 2021-10-05

**Authors:** Karoline B. Rypdal, Pugazendhi M. Erusappan, A. Olav Melleby, Deborah E. Seifert, Sheryl Palmero, Mari E. Strand, Theis Tønnessen, Christen P. Dahl, Vibeke Almaas, Dirk Hubmacher, Suneel S. Apte, Geir Christensen, Ida G. Lunde

**Affiliations:** 1 Institute for Experimental Medical Research, Oslo University Hospital and University of Oslo, Building 7, 4th floor, Kirkeveien 166, 0407 Oslo, Norway; 2 KG Jebsen Cardiac Research Center and Center for Heart Failure Research, University of Oslo, Oslo, Norway; 3 Section of Physiology, Department of Molecular Medicine, Institute for Basic Medical Sciences, University of Oslo, Oslo, Norway; 4 Department of Biomedical Engineering, Cleveland Clinic Lerner Institute, Cleveland, OH USA; 5 Department of Cardiothoracic Surgery, Oslo University Hospital Ullevaal, Oslo, Norway; 6 Department of Cardiology, Oslo University Hospital Rikshospitalet, Oslo, Norway; 7 Orthopaedic Research Laboratories, Leni & Peter W. May Department of Orthopaedics, Icahn School of Medicine at Mount Sinai, New York, NY USA

**Keywords:** Cell biology, Molecular biology, Cardiology, Medical research, Molecular medicine

## Abstract

Fibrosis accompanies most heart diseases and is associated with adverse patient outcomes. Transforming growth factor (TGF)β drives extracellular matrix remodelling and fibrosis in the failing heart. Some members of the ADAMTSL (a disintegrin-like and metalloproteinase domain with thrombospondin type 1 motifs-like) family of secreted glycoproteins bind to matrix microfibrils, and although their function in the heart remains largely unknown, they are suggested to regulate TGFβ activity. The aims of this study were to determine ADAMTSL2 levels in failing hearts, and to elucidate the role of ADAMTSL2 in fibrosis using cultured human cardiac fibroblasts (CFBs). Cardiac ADAMTSL2 mRNA was robustly increased in human and experimental heart failure, and mainly expressed by fibroblasts. Over-expression and treatment with extracellular ADAMTSL2 in human CFBs led to reduced TGFβ production and signalling. Increased ADAMTSL2 attenuated myofibroblast differentiation, with reduced expression of the signature molecules α-smooth muscle actin and osteopontin. Finally, ADAMTSL2 mitigated the pro-fibrotic CFB phenotypes, proliferation, migration and contractility. In conclusion, the extracellular matrix-localized glycoprotein ADAMTSL2 was upregulated in fibrotic and failing hearts of patients and mice. We identified ADAMTSL2 as a negative regulator of TGFβ in human cardiac fibroblasts, inhibiting myofibroblast differentiation and pro-fibrotic properties.

## Introduction

Fibrosis is a hallmark feature of heart failure, a leading cause of morbidity and mortality worldwide, and the degree of cardiac fibrosis is a strong predictor of poor outcomes in patients^1–4^. Cardiac fibrosis results from dysregulated deposition of extracellular matrix (ECM) molecules by activated cardiac fibroblasts (CFBs), termed myofibroblasts, leading to tissue stiffening^5–7^. Transforming growth factor (TGF)β is a central signalling molecule in cardiac development and disease, and a major driver of cardiac myofibroblast differentiation and fibrosis^8–10^. Inactive TGFβ, bound to latent TGFβ binding proteins (LTBPs), is stored in the ECM as the large latent complex (LLC), tethered to microfibrils^11,12^. Release from the inactive complex is required for TGFβ activation and initiation the pro-fibrotic response^10^. Thus, identification of molecular players governing TGFβ signalling in the heart is essential to develop novel treatment strategies that can counteract cardiac fibrosis.

Members of the ADAMTSL (a disintegrin-like and metalloproteinase domain with thrombospondin type 1 motifs-like) family of secreted glycoproteins structurally resemble ADAMTS proteases, but lack enzymatic activity, as they do not have a protease domain^13,14^. Some ADAMTS proteases have important roles in the heart^15–17^, but little is known about the role of ADAMTSLs. Emerging data suggests that they can bind and regulate fibrillin microfibrils^18–20^, major ECM components that control TGFβ bioavailability. Recessive loss-of-function *ADAMTSL2* mutations cause geleophysic dysplasia (GD), an inherited connective tissue disorder resulting in severe musculoskeletal, pulmonary, and cardiac anomalies, with increased TGFβ levels and activity observed in patient-derived skin fibroblasts^21,22^. In beagles, an *ADAMTSL2* founder mutation causes Musladin-Lueke Syndrome with severe skin and intermuscular fibrosis^23^. *Adamtsl2*−/− mice fail to survive past birth, likely as a result of lung anomalies associated with bronchial fibrillin microfibril accumulation, and have cardiac developmental defects^24^. Collectively, these data suggest that ADAMTSL2 regulates ECM deposition and TGFβ signalling and may thus have an important role in cardiac fibrosis and heart failure.

In the present study, we investigated expression of ADAMTSLs in heart failure, and identified novel functions of ADAMTSL2 in CFBs. Specifically, we found that ADAMTSL2 was robustly up-regulated in clinical and experimental heart failure, and ADAMTSL2 was predominantly expressed by CFBs. In cultured foetal and adult human CFBs, ADAMTSL2 negatively regulated TGFβ signalling, and attenuated myofibroblast differentiation and pro-fibrotic properties of CFBs.

## Results

### ADAMTSL1-5 and Papilin are up-regulated in hearts of mice with fibrosis and failure

**Figure 1:**
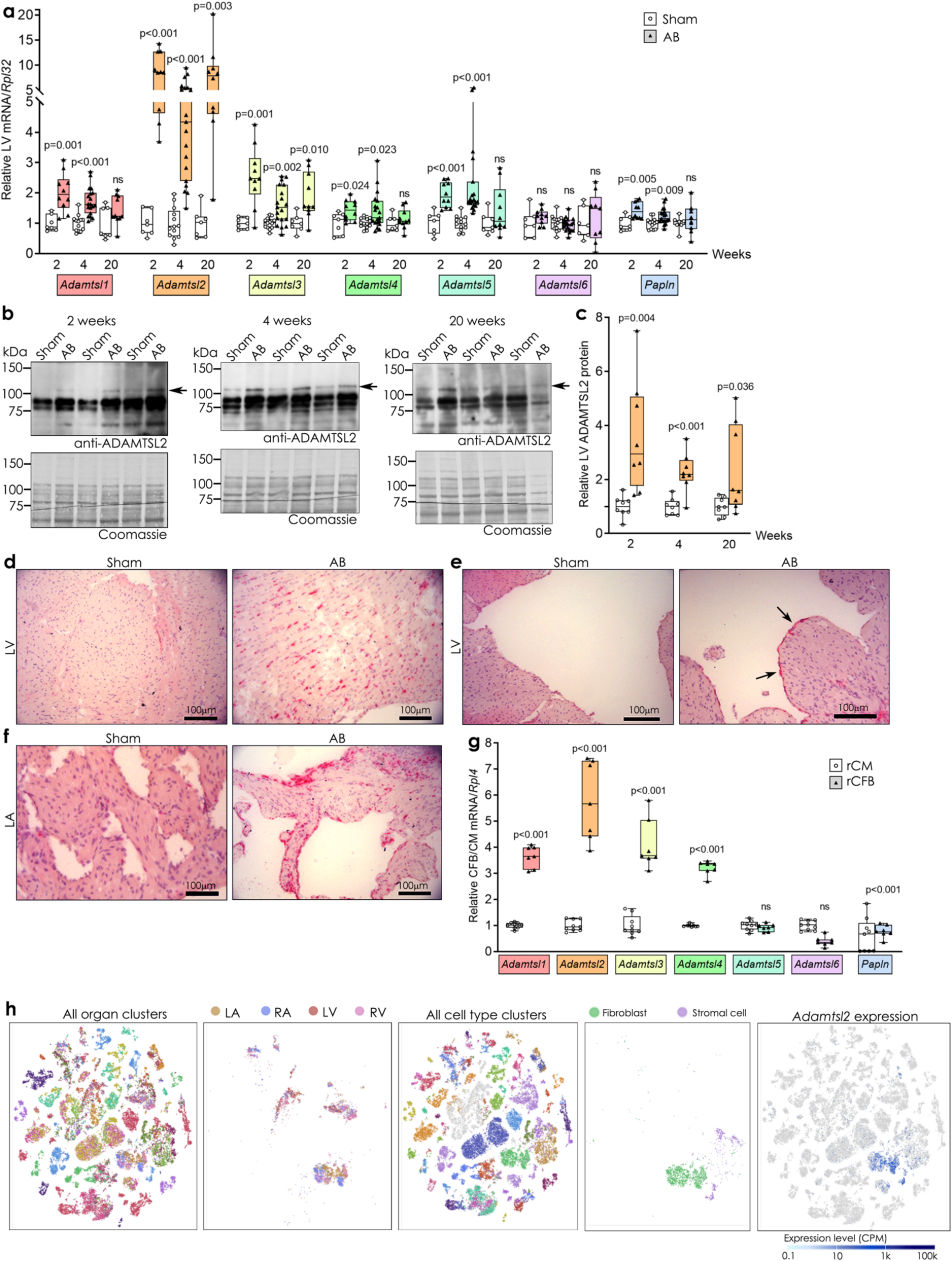
ADAMTSL mRNA is up-regulated in fibrotic, failing mouse hearts, and ADAMTSL2 is produced by cardiac fibroblasts. (**a**) mRNA levels of the seven *ADAMTSL* genes in left ventricles (LV) of mice 2, 4 and 20 weeks post aortic banding (AB) or sham surgery (n = 7–13 sham and 10–19 AB mice per time point). Phenotypic characteristics have been published previously^25^. Gene expression was normalized to *Rpl32*. (**b**) Representative immunoblots of ADAMTSL2 (arrow) in LVs at 2, 4 and 20 weeks post-AB compared to sham controls. Uncropped blots are available in Supplementary figure I. (**c**) Quantification of blots (n = 8 sham and n = 8 AB mice per time point). (**d–f**) Representative in situ hybridization images of *Adamtsl2* mRNA in cardiac tissue (LV and left atrium (LA)), from AB or sham mice at 4 weeks post surgery (n = 3 sham and n = 4 AB mice). *Adamtsl2* expression (bright red dots) is seen in the LV wall (d) regions of the ventricular endocardium (e, arrows), and LA wall and endocardium (f). Hematoxylin (purple) was used as nuclear counterstain. (**g**) mRNA levels of the ADAMTSL family in cardiac fibroblast (rCFB) and cardiomyocyte (rCM) primary cultures, isolated from 1–3 days old neonatal rats (n = 3 isolations with n = 60 hearts per isolation). Gene expression was normalized to *Rpl4.* Data (a, c, g) are mean ± min/max values and statistical analysis was performed using the Student *t*-test. (**h**) Data mined from EMBL-EBI Single Cell Expression Atlas showing single-cell RNA sequencing of 20 different mouse organs and tissues from n = 3 female and n = 4 male 10–15 week old mice^26^ (left-hand panel). *Adamtsl2* expression (right hand panel) co-clustered with cardiac cells from the right atrium (RA), LA, right ventricle (RV) and LV (second panel from left), and with fibroblasts and stromal cells (fibroblast lineage) (centre and second panel from the right). Scale bar = expression level as counts per million reads mapped (CPM).

To understand the role of ADAMTSL proteins in heart failure, we determined ADAMTSL gene expression in left ventricles (LV) of mice subjected to AB for two, four and 20 weeks. The resulting cardiac phenotypes were previously reported^25^, and in summary show concentric hypertrophic remodelling at two and four weeks post-AB, where the heart muscle is thickened, and end-stage dilated heart failure at 20 weeks, with thinning of the heart muscle, compared to sham-operated controls. Cardiac fibrosis (increased collagen expression and extracellular deposition) and increased TGFβ signalling was present at all three time-points, with increased α-smooth muscle actin (α-SMA) observed at two weeks^25^. We found that all members of the *Adamtsl* family were upregulated after AB, except *Adamtsl6,* which was unchanged. *Adamtsl2* showed the highest up-regulation with a four–eightfold increase during hypertrophic remodelling, and an eightfold increase at end-stage cardiac dilatation (Fig. 1a), suggesting a potential role in fibrosis and heart failure. Thus, we focused our efforts on ADAMTSL2.

Immunoblotting for ADAMTSL2 in LV protein extracts was consistent with *Adamtsl2* mRNA induction, showing a 2-3.5-fold increase in full-length protein at two, four and 20 weeks post-AB (Fig. 1b,c). In situ hybridization (ISH) in heart sections showed that *Adamtsl2* mRNA expression was increased in the LV and left atrium (LA) after AB, compared to modest expression of *Adamtsl2* in the sham-operated mice (Fig. 1d–f). Importantly, increased *Adamtsl2* expression in AB mice was seen between cardiomyocytes (CM) in the LV wall (Fig. 1d), in regions of the LV endocardium (Fig. 1e, arrows) and the LA endocardium (Fig. 1f), suggesting an increase in ADAMTSL2 expression in cell types other than CMs across the heart.

### ADAMTSL2 is mainly expressed by cardiac fibroblasts in the ventricular wall

As two of the dominant cell types in the ventricular wall, we investigated expression of the ADAMTSLs in primary cultures of isolated CMs and CFBs from neonatal rat hearts. The purity of the cultures was confirmed by the cardiomyocyte marker Troponin-I (*Tnni3*), the endothelial cell marker von Willebrand Factor (*Vwf*), and the fibroblast markers type I Collagen (*Col1a2*), Periostin (*Postn*), and α-SMA (*Acta2*) (Supplementary Fig. S1a–c). *Adamtsl1-4* were mainly expressed by CFBs, with fivefold higher *Adamtsl2* expression in CFBs compared to CMs, while *Papln* expression was higher in CMs (Fig. 1g). As endothelial cells were enriched in the CM cultures, our data suggest CFBs as the main cellular source of ADAMTSL2 in the ventricle. In line with this, data mined from published single-cell RNA sequencing studies^26^ showed that *Adamtsl2* was specifically expressed by fibroblasts and stromal cells (fibroblast lineage) in the mouse heart (Fig. 1h). Combined, these results indicate that CFBs constitute the main source of ADAMTSL2 in the heart.

### ADAMTSL2 over-expression inhibits TGFβ signalling in human cardiac fibroblasts

**Figure 2:**
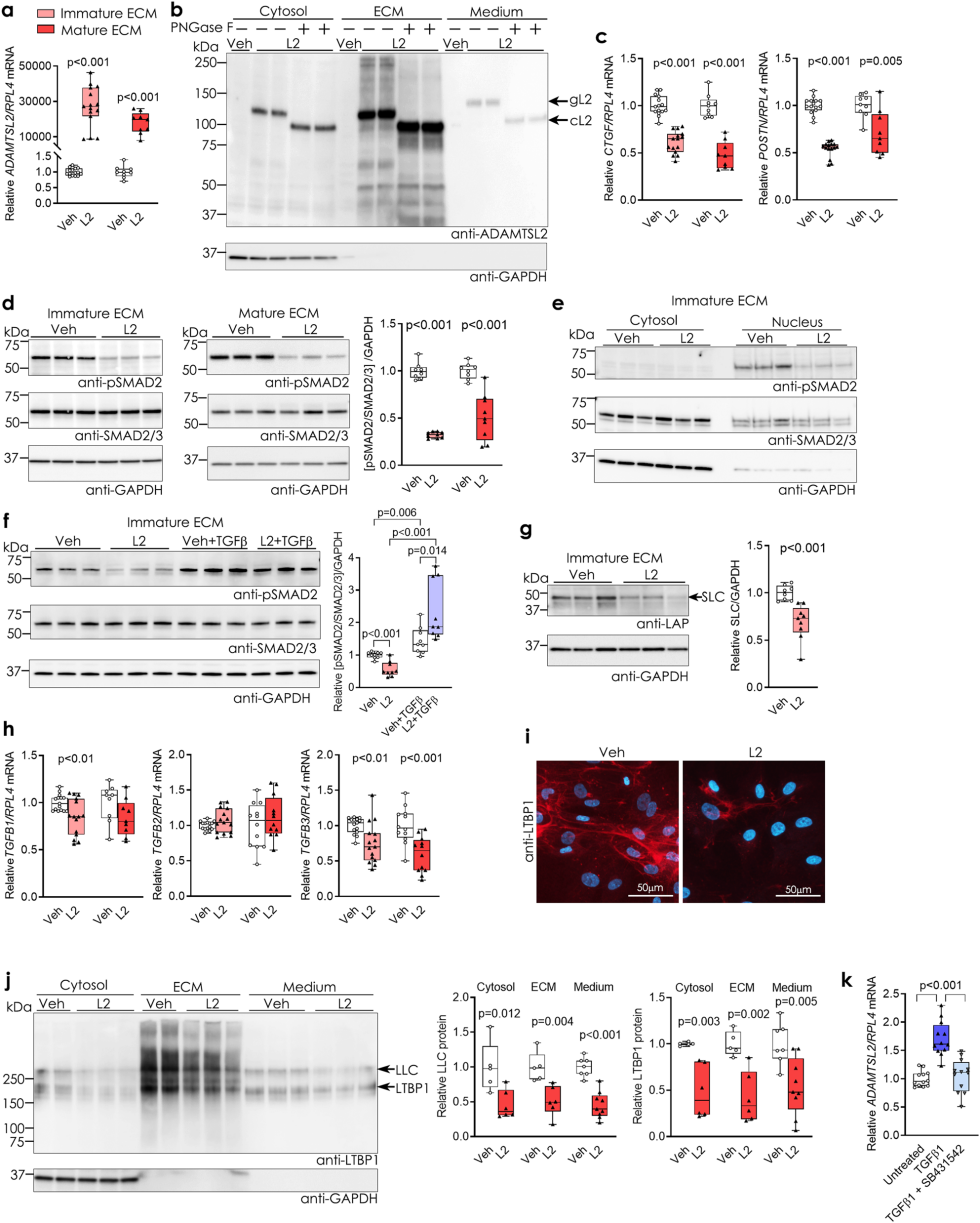
ADAMTSL2 inhibits TGFβ signalling in human cardiac fibroblasts. Human foetal cardiac fibroblasts (hfCFBs) were cultured for four or seven days (see Supplementary Fig. S3a), forming an immature or mature extracellular matrix (ECM), and transduced with ADAMTSL2 (L2) or vehicle control (Veh) adenoviruses on day one or four, respectively. Data represent experiments from three different cell passages. (**a**) Successful over-expression of ADAMTSL2 in L2 vs. Veh. (**b**) Immunoblot of full-length, glycosylated ADAMTSL2 (gL2)^47^, and deglycosylated ADAMTSL2 (cL2), in L2 and not in Veh cell fractions. (**c**) mRNA levels of TGFβ downstream targets, i.e. connective tissue growth factor (*CTGF)* and periostin (*POSTN),* in L2 vs. Veh (n = 9–15). (**d**) Representative immunoblots and quantification of phosphorylated (pSMAD2) and total SMAD2/3 in L2 vs. Veh (n = 9). (**e**) Representative immunoblots and quantification of pSMAD2 and total SMAD2/3 in cytosol and nucleus cell fractions of L2 vs. Veh (n = 9). (**f**) Representative immunoblots and quantification of pSMAD2 and total SMAD2/3 in lysates from L2 vs. Veh with and without treatment with active TGFβ for 24 h (n = 9). (**g**) Representative immunoblot and quantification of latency associated peptide (LAP) in lysates from L2 vs. Veh, showing the small latent complex (SLC) consisting of TGFβ and LAP (n = 9). (**h**) mRNA levels of *TGFB1, TGFB2* and *TGFB3,* in L2 vs. Veh (n = 9–15). (**i**) Representative immunocytochemistry images of latent TGFβ-binding protein (LTBP1, red) and DAPI (blue) in L2 vs. Veh (n = 3). (**j**) Representative immunoblot and quantification of LTBP1 and the large latent complex (LLC), in cytosol, ECM and medium of L2 vs Veh with mature ECM (n = 5–9 per group). (**k**) *ADAMTSL2* mRNA levels in untreated hfCFBs, hfCFBs treated with recombinant TGFβ and hfCFBs treated with TGFβ-SMAD inhibitor SB431542. Uncropped blots are available in Supplementary figure II. GAPDH was used as loading control (**b**, **d**, **e**, **f**, **g**, **j**). mRNA was normalized to *RPL4* (**a**, **c**, **h**, **k**). Data are mean ± min/max and statistical analysis was performed using the Student *t*-test vs. respective controls (**a**, **c–j**), and one-way ANOVA with Tukey’s multiple comparisons test (**k**).

To determine the role of ADAMTSL2 in CFBs, gain-of-function in vitro studies were performed. We over-expressed ADAMTSL2 using replication-deficient adenovirus 5 containing *ADAMTSL2* (L2) or vehicle control (Veh) in hfCFBs. Successful over-expression was confirmed by increased *ADAMTSL2* mRNA (Fig. 2a) and increased full-length, glycosylated protein in the cell cytosol, ECM and medium (Fig. 2b). Loss-of-function studies were considered, but deemed unsuitable, as basal ADAMTSL2 expression was barely detectable in our cultured hfCFBs (Supplementary Fig. S2a-c), in line with negligible detection of *ADAMTSL2* mRNA in cultured human dermal fibroblasts from deceased organ donors in the GTEx project (dbGaP Acession phs000424.v8.p2) (Supplementary Fig. S2d).

Effects of increased ADAMTSL2 levels were investigated in hfCFBs at two stages of ECM maturation i.e. cells were transduced at day one of a four-day protocol (immature ECM) or at day four of a seven-day protocol (mature ECM) (Supplementary Fig. S3a). Matrix proteins showed differential gene expression in the two protocols (Supplementary Fig. S3b-c), and importantly, greater ECM accumulation was evident at seven days (Supplementary Fig. S3d). More LLC was incorporated into the mature ECM (Supplementary Fig. S3e), and the cells became less proliferative at this stage (Supplementary Fig. S3f.). Myofibroblast differentiation occurred within the first four days in culture, as evident from the peak expression at four days (Supplementary Fig. S3g) but persisted through seven days (Supplementary Fig. S3d,g). TGFβ signalling was comparable in the two protocols (Supplementary Fig. S3h). Thus, the seven-day protocol was used to investigate the role of ADAMTSL2 in a mature ECM, while the four-day protocol was used to study its role in a developing, immature ECM.

First, we assessed whether increased ADAMTSL2 affected TGFβ signalling in CFBs. We found that mRNA levels of direct downstream targets of TGFβ signalling in the heart, connective tissue growth factor (*CTGF*) and periostin (*POSTN*)^27,28^*,* were reduced 30–50% in L2 cells in both culture protocols (Fig. 2c). Canonical TGFβ signalling is mediated mainly through phosphorylation of SMAD2/3 transcription factors, which translocate to the nucleus^8^, and we found that L2 decreased SMAD2 phosphorylation (pSMAD) 50–70% in the two protocols (Fig. 2d). Correspondingly, the pSMAD2/3 complex translocated to the nucleus in Veh, but less so in L2 cells (Fig. 2e). Thus, our data demonstrate that ADAMTSL2 caused reduced TGFβ signalling in CFBs.

Next, we addressed how ADAMTSL2 reduced TGFβ signalling. To assess whether ADAMTSL2 inhibited the active, free form of TGFβ directly, or its interaction with the TGFβ receptor (TGFBR), recombinant, active TGFβ1 was added to Veh and L2 cells. Although L2 cells had lower levels of pSMAD at baseline, we found that TGFβ treatment increased pSMAD in both Veh and L2 cells, with a higher pSMAD increase in L2 cells relative to respective baseline levels (Fig. 2f). This demonstrated that ADAMTSL2 did not inhibit active TGFβ signalling, and indicate that the effect of ADAMTSL2 on TGFβ is upstream of active, released TGFβ. Immunoblotting for the small latency complex (SLC), consisting of TGFβ bound to latency associated peptide (LAP), showed a 35% reduction of SLC in L2 cells (Fig. 2g), suggesting reduced TGFβ production. Furthermore, mRNA expression of *TGFB1* was reduced to 85% of control in the immature ECM culture and *TGFB3* to 60–75% of controls in the immature and mature ECM cultures (Fig. 2h). In line with reduced production of TGFβ, we found that LTBP1 and the LLC were reduced in L2 cell fractions from the cytosol, ECM, and medium of mature ECM hfCFB cultures. (Fig. 2i–j). There was no change in gene expression of the three LTBP isoforms that bind TGFβ, namely LTBP1, LTBP3 and LTBP4 (Supplementary Fig. S4a-c), indicating protein-level regulation. Finally, CFB treatment with recombinant TGFβ1 resulted in a 1.6-fold increase in *ADAMTSL2* mRNA levels that was returned to baseline upon co-treatment with the TGFβ-SMAD inhibitor SB431542 (Fig. 2k). These results suggest that ADAMTSL2 is under transcriptional control of TGFβ signalling, and that ADAMTSL2 regulates TGFβ as part of a negative feedback loop.

### ADAMTSL2 alters expression levels of ECM and ECM-associated genes in cardiac fibroblasts

**Figure 3:**
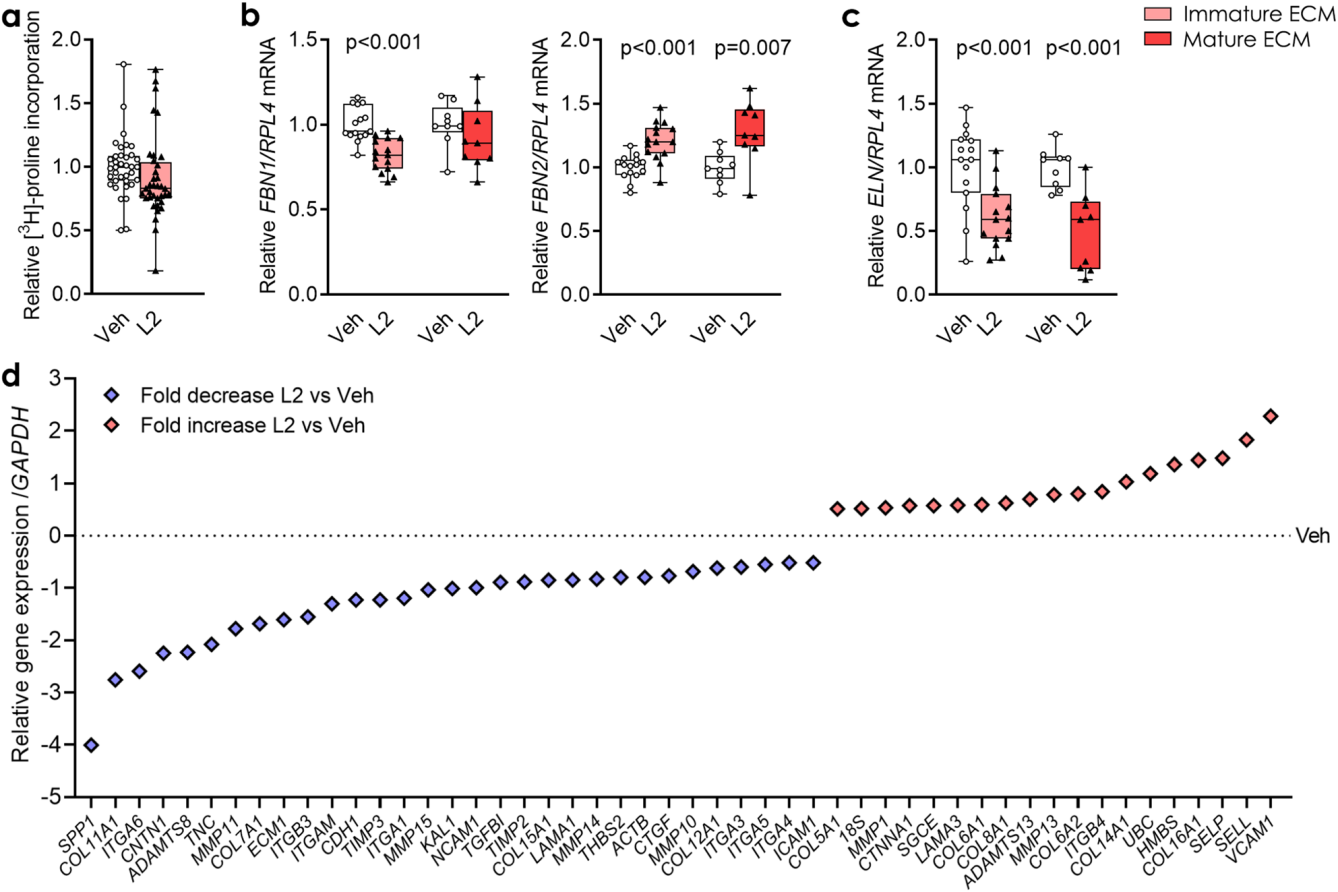
ADAMTSL2 alters expression of ECM and ECM-associated genes in human cardiac fibroblasts. Human foetal cardiac fibroblasts were cultured for four or seven days (see Supplementary Fig. S3a), forming an immature or mature extracellular matrix (ECM), and transduced with ADAMTSL2 (L2) or vehicle control (Veh) adenoviruses on day one or four, respectively. Data represent experiments from three different cell passages. (**a**) Incorporation of [^3^H]-proline representing total collagen synthesis in L2 vs. Veh (n = 36). (**b**, **c**) *FBN1*, *FBN2* and *ELN* mRNA levels in L2 vs. Veh (n = 9–15). Gene expression was normalized to *RPL4* (**b**, **c**). Data are presented as mean ± min/max values and statistical analysis was performed using the Student *t*-test vs. respective controls. (**d**) Gene expression array of 84 ECM and adhesion molecule genes showing 50 differentially expressed genes, ± 0.5-fold in L2 vs. Veh (pools of n = 3–6). Gene expression was normalized to *GAPDH* and Veh and presented as –ΔΔCT values.

We next examined whether ADAMTSL2 over-expression altered production of major ECM constituents. As the classical definition of cardiac fibrosis is the accumulation of fibrillar collagens^5^, we quantified the amount of newly synthesized collagen in immature ECM hfCFB cultures. However, we found no difference in collagen synthesis (Fig. 3a). Thus, despite inhibiting TGFβ signalling, ADAMTSL2 did not affect levels of structural collagens in cultured hfCFBs.

To address whether ADAMTSL2 regulated ECM microfibrils we measured the expression of fibrillin-1 (*FBN1)*, fibrillin-2 (*FBN2*) and tropoelastin (*ELN)* in L2 cells. The mRNA level of *FBN1* was reduced to 80% of control in the immature ECM culture, and *ELN* to 40–60% of controls in both cultures, while mRNA levels of *FBN2* were increased 1.2-fold in both cultures (Fig. 3b,c). These results suggest that ADAMTSL2 may regulate the composition of tissue microfibrils in the cardiac ECM.

Using array expression analysis of 84 ECM and cell adhesion genes (see Supplementary Fig. S5), we found 50 differentially expressed genes ± 0.5-fold or more (31 down-regulated/19 up-regulated) in L2 cells (Fig. 3d). Proteins involved in cell adhesion, proliferation and spreading, such as integrin β3 (*ITGB3*), contactin-1 (*CNTN1*)*,* E-cadherin (*CDH1*) and tenascin C (*TNC*) were down-regulated, while adhesion molecules related to inflammation, vascular cell adhesion molecule-1 (*VCAM1*), L-selectin (*SELL*) and P-selectin (*SELP*), were among the most up-regulated. Collagen degrading enzymes such as matrix metalloproteinase 1 and 13 (*MMP1, MMP13*), as well as non-fibrillar collagens (*COL6A1, COL6A2, COL8A1, COL14A1, COL16A1*) were also among the up-regulated. Strikingly, osteopontin mRNA (*SPP1*) was down-regulated to 6% of controls (Fig. 3d). As both TGFβ and osteopontin signalling are required myofibroblast differentiation^8,29^, this indicate that ADAMTSL2 inhibited myofibroblast differentiation.

### ADAMTSL2 inhibits myofibroblast differentiation and regulates cardiac fibroblast function

**Figure 4:**
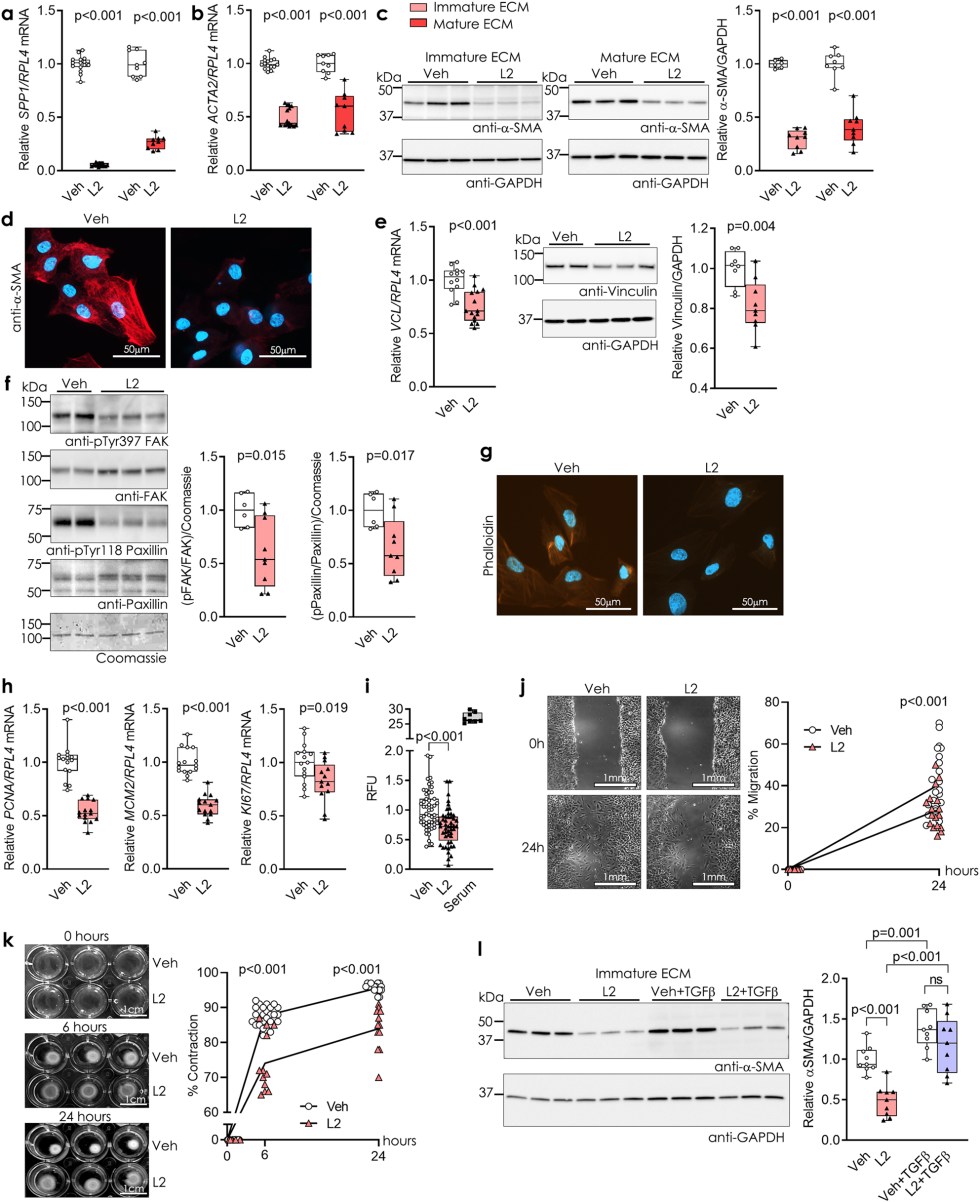
ADAMTSL2 inhibits myofibroblast differentiation and directs cardiac fibroblast function. Human foetal cardiac fibroblasts were cultured for four or seven days (see Supplementary Fig. S3a), forming an immature or mature extracellular matrix (ECM), and transduced with ADAMTSL2 (L2) or vehicle control (Veh) adenoviruses on day one or four, respectively. Data represent experiments from three different cell passages. (**a**) mRNA levels of osteopontin (*SPP1)* and (**b**) α-smooth muscle actin (α-SMA, *ACTA2)* in L2 vs. Veh (n = 9–15). (**c**) Representative immunoblot and quantification of α-SMA in lysates from L2 vs. Veh (n = 9). (**d**) Representative immunocytochemistry images of α-SMA (red) and DAPI (blue) in L2 vs. Veh (n = 3). (**e**) mRNA levels of vinculin (*VCL*) and representative immunoblot and quantification of vinculin in L2 vs. Veh (n = 9). (**f**) Representative immunoblots and quantifications of phosphorylated and total focal adhesion kinase (pTyr925 FAK and FAK) and phosphorylated (pTyr118) and total paxillin in lysates from L2 vs. Veh (n = 9). (**g**) Representative immunocytochemistry images of F-actin stress fibres (phalloidin, orange) and DAPI (blue) in L2 vs. Veh (n = 3). (**h**) mRNA levels of proliferating cell nuclear antigen (*PCNA*), minichromosome maintenance protein 2 (*MCM2*) and marker of proliferation Ki-67 (*KI67*) in L2 vs. Veh (n = 15). (**i**) EdU incorporation shown as relative fluorescence units (RFU) in L2 vs Veh (n = 48–52), serum was used as positive control. (**j**) Cell migration shown as % of initial scratch area in cell monolayer after 24 h, in L2 vs. Veh (n = 29). (**k**) Representative images and quantification of collagen gel contraction as % contraction of initial gel area, measured at 6 and 24 h, of L2 vs. Veh. (**l**) Representative immunoblots and quantification of α-SMA lysates from L2 vs. Veh with and without treatment with active TGFβ for 24 h (n = 9). Uncropped blots are available in Supplementary figure III. Gene expression was normalized to *RPL4* (**a**, **b**, **e**, **h**). GAPDH (**c**, **e**, **l**) and Coomassie blue staining (f) was used as protein loading control. Data are mean ± min/max values and statistical analysis was performed using the Student *t*-test vs. respective controls, or two-way repeated measures ANOVA with the Geisser-Greenhouse correction (**k**).

We confirmed negative osteopontin regulation in L2 cells, and found that *SPP1* was reduced to 5% and 30% of controls, in our immature and mature ECM hfCFB cultures, respectively (Fig. 4a). We next investigated myofibroblast markers and found that expression of *ACTA2,* encoding α-SMA, was reduced 50% in both culture protocols (Fig. 4b). Correspondingly, immunoblotting showed a 30–40% reduction of α-SMA protein (Fig. 4c), and a strong reduction in α-SMA immunostaining (Fig. 4d). These results indicate that ADAMTSL2 inhibits cardiac myofibroblast differentiation.

Upon further investigation of characteristic myofibroblast properties we found that increased ADAMTSL2 resulted in reduced vinculin mRNA and protein levels (Fig. 4e), reduced focal adhesion kinase (FAK) activation and phosphorylation of paxillin (Fig. 4f), and impaired stress fibre formation, illustrated by reduced phalloidin immunostaining (Fig. 4g). Levels of the cell proliferation markers proliferative cell nuclear antigen (*PCNA*), mini-chromosome maintenance protein 2 (*MCM2*) and marker of proliferation Ki-67 (*KI67*) were reduced to 50–80% of controls (Fig. 4h), indicating that ADAMTSL2 inhibits CFB proliferation. In line with this, incorporation of EdU during DNA synthesis was reduced in L2 cells, corresponding to reduced proliferation (Fig. 4i). A scratch assay was performed to assess cell migration, and L2 cells showed reduced migration across the gap compared to Veh (Fig. 4j). As the hallmark feature of myofibroblasts, we assessed whether ADAMTSL2 affected CFB contraction, and found that the ability to contract collagen gels was impaired in L2 cells (Fig. 4k).

To assess whether myofibroblast differentiation could be rescued by treatment with TGFβ, recombinant, active TGFβ1 was added to the Veh and L2 cultures. Protein expression of α-SMA increased in both conditions, relative to respective baseline levels, following treatment (Fig. 4l). This indicates that ADAMTSL2 may inhibit myofibroblast differentiation through inhibition of TGFβ. Collectively, these data show that ADAMTSL2 modulated CFBs towards a less myofibroblastic and less fibrotic phenotype.

### Extracellular ADAMTSL2 inhibits TGFβ signalling in human cardiac fibroblasts

**Figure 5:**
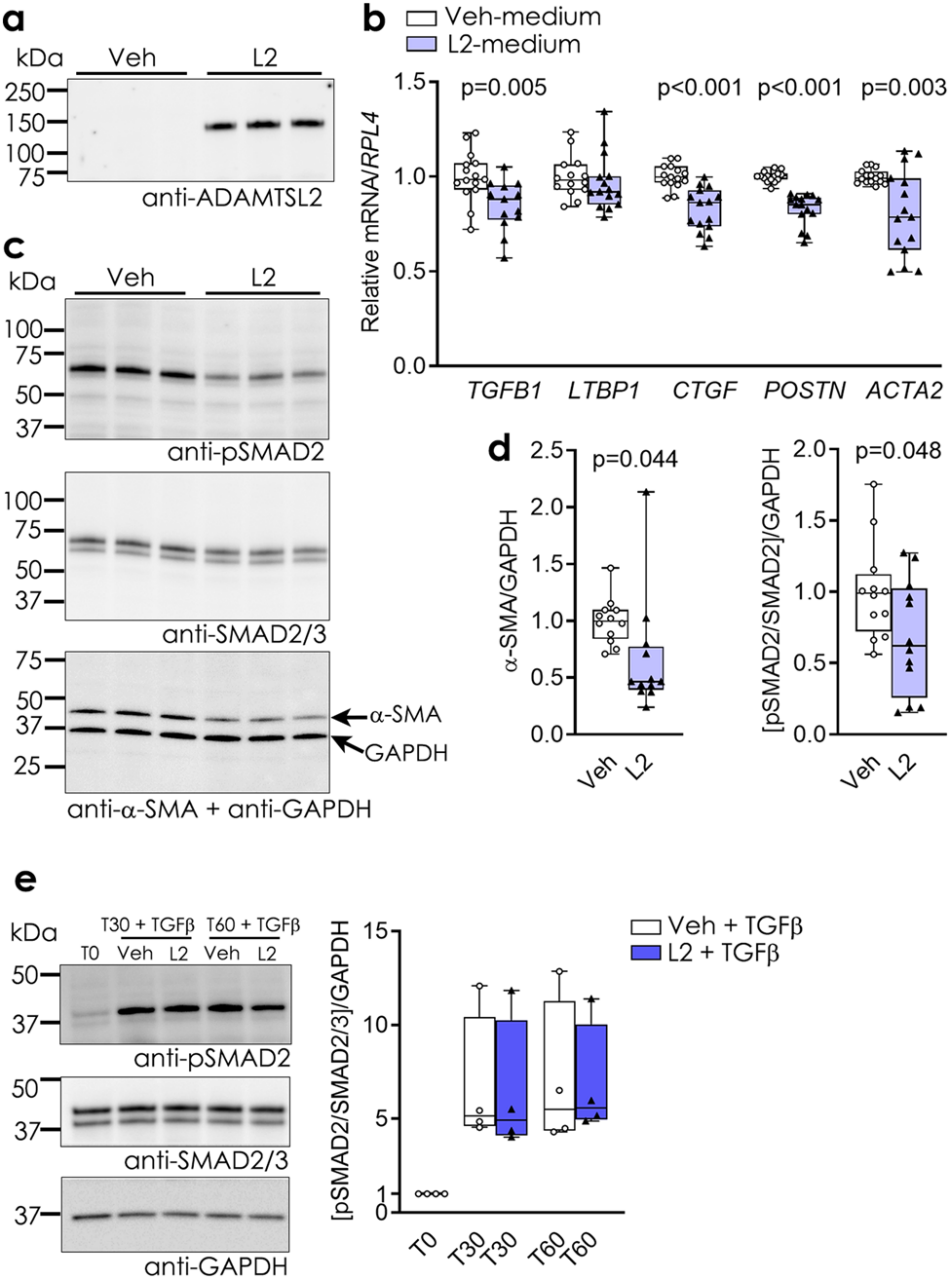
Extracellular ADAMTSL2 inhibits TGFβ signalling and myofibroblast differentiation in human cardiac fibroblasts. Human foetal cardiac fibroblasts (hfCFBs) treated with conditioned medium harvested from hfCFBs transduced with ADAMTSL2 (L2-medium) or vehicle control (Veh-medium, see Supplementary Fig. S3a). Data represent experiments from 3–5 different cell passages (n = 15). (**a**) Immunoblot of ADAMTSL2 in L2-medium and Veh-medium. (**b**) mRNA levels of transforming growth factor (TGF)β 1 (*TGFB1*)*,* latent TGFβ binding protein 1 (*LTBP1*), connective tissue growth factor (*CTGF*), periostin (*POSTN*), and α-smooth muscle actin (α-SMA, encoded by *ACTA2*). (**c**) Representative immunoblots of phosphorylated (pSMAD2), total SMAD2/3 and α-SMA in whole-cell protein lysates and (**d**) quantification of blots. (**e**) Representative immunoblots and quantification of pSMAD2 and total SMAD2/3 in lysates from hfCFBs treated with Veh- or L2-medium, which was pre-incubated with active, TGFβ. hfCFBs were treated for 0 (T0), 30 (T30) or 60 (T60) minutes. Uncropped blots are available in Supplementary figure IV. Gene expression was normalized to *RPL4.* GAPDH was used as intracellular protein loading control. Data are presented as mean ± min/max values and statistical analysis was performed using the Student *t*-test vs. respective controls.

To determine whether the observed effects of ADAMTSL2 over-expression were due to ADAMTSL2’s function in the extracellular environment, hfCFBs were treated with conditioned medium from L2 cells (L2-medium), which had demonstrably higher levels of ADAMTSL2 (Fig. 5a), or controls (Veh-medium). In line with ADAMTSL2 over-expression, treatment with L2-medium resulted in reduced expression of *TGFB1, CTGF, POSTN* and *ACTA2*, but still unaltered *LTBP1* expression, compared to Veh-medium (Fig. 5b). Furthermore, cells treated with L2-medium showed reduced phosphorylation of SMAD2 and reduced levels of α-SMA (Fig. 5c–d). To determine whether extracellular ADAMTSL2 would affect the active, free form of TGFβ, Veh- and L2-medium was pre-incubated with recombinant TGFβ and added to untreated hfCFBs. Both conditions resulted in similar increase in pSMAD after 30 and 60 min of treatment, confirming that ADAMTSL2 does not directly bind and inhibit free, active TGFβ (Fig. 5e). Thus, these data confirmed the findings from over-expression of ADAMTSL2, and showed that hfCFBs respond to increased extracellular ADAMTSL2 with reduced TGFβ activity and myofibroblast differentiation.

### ADAMTSL2 is increased in human hearts with fibrosis and heart failure

**Figure 6:**
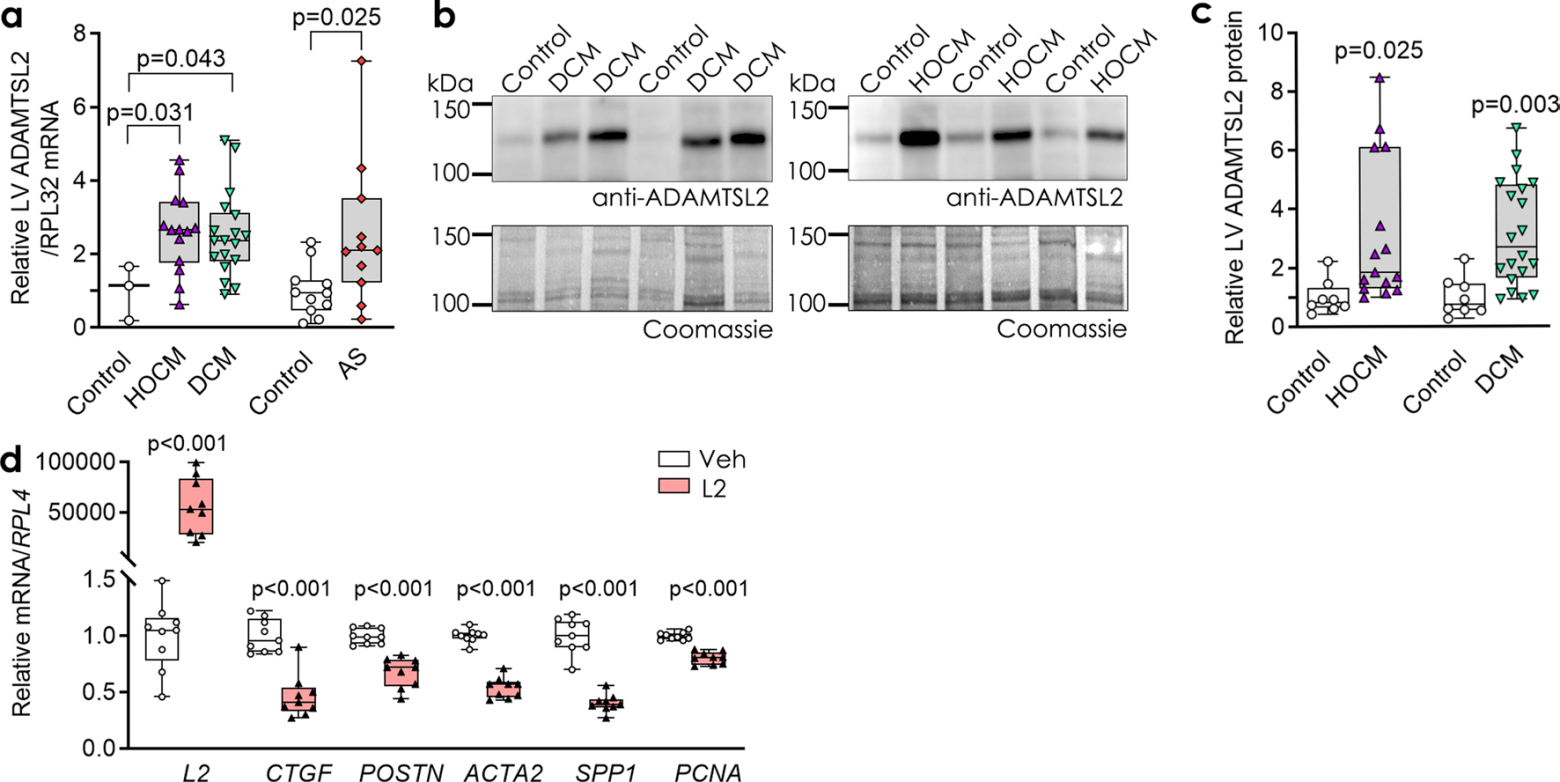
ADAMTSL2 is increased in left ventricles from patients with heart failure and reduces expression of pro-fibrotic genes in adult human cardiac fibroblasts. (**a**) *ADAMTSL2* mRNA and (**b**, **c**) protein levels in left ventricles of patients with hypertrophic obstructive cardiomyopathy (HOCM, n = 15), dilated cardiomyopathy (DCM, n = 20), and aortic stenosis (AS, n = 11) vs. respective controls. Gene expression was normalized to *RPL32* (**a**) and Coomassie blue staining was used as protein loading control (**b**). Uncropped blots are available in Supplementary figure V. (**d**) Human adult cardiac fibroblasts were transduced with ADAMTSL2 (L2) or control vehicle (Veh) adenoviruses. The graph shows mRNA levels of connective tissue growth factor (*CTGF*), periostin (*POSTN*), α-smooth muscle actin (*ACTA2*), osteopontin (*SPP1*) and proliferating cell nuclear antigen (*PCNA*) in L2 vs. Veh. Data represent experiments from three different cell passages (n = 9) and are presented as mean ± min/max values. Statistical analysis was performed using the Student *t*-test vs. respective controls.

Finally, from a translational perspective, we investigated ADAMTSL2 levels in hearts of patients with heart failure and assessed the effects of ADAMTSL2 in human adult CFBs (haCFBs). From the GTEx project of deceased organ donors, it was evident that ADAMTSL2 was expressed in human hearts, both in left ventricle and atrial appendage (Supplementary Fig. S2d and Table S3). We determined *ADAMTSL2* mRNA levels in myocardial biopsies from three cohorts of patients, namely aortic stenosis (AS), hypertrophic obstructive cardiomyopathy (HOCM) and dilated cardiomyopathy (DCM) vs. respective controls. Patient characteristics were reported previously, with hypertrophic remodelling, fibrosis and heart failure with preserved ejection fraction (HFpEF) in AS and HOCM, and dilated heart failure with reduced ejection fraction (HFrEF) and fibrosis in DCM patients^30–32^. Importantly, *ADAMTSL2* mRNA was increased 2.5-fold in LVs of HOCM, DCM and AS patients (Fig. 6a). Immunoblotting of LV extracts confirmed increased full-length ADAMTSL2 protein in biopsies from HOCM and DCM patients (Fig. 6b-c and Supplementary Fig. S6). Biopsies from the AS patients were exclusively used for mRNA analyses due to small sample size. Finally, ADAMTSL2 was successfully over-expressed in haCFBs in a four-day protocol similar to hfCFBs (see Supplementary Fig. S3), and we found that mRNA levels of *CTGF*, *POSTN*, *ACTA2, SPP1* and *PCNA* were reduced compared to controls (Fig. 6d). Thus, the increased levels of Adamtsl2 observed in experimental heart failure was confirmed in human heart failure, and our findings from hfCFBs translated to ventricular haCFBs.

## Discussion

The present work places the ADAMTSL family of ECM glycoproteins on the map of influential molecules in the failing heart. We found that six out of seven Adamtsl proteins were up-regulated in hearts of mice with AB-induced heart failure and fibrosis. In particular, ADAMTSL2 mRNA and protein levels were robustly increased in both experimental and clinical heart failure. In the heart, *Adamtsl2* was mainly expressed by CFBs, and increased levels of ADAMTSL2 in human CFBs in vitro, through viral over-expression or exposure to ADAMTSL2-containing conditioned medium, resulted in reduced TGFβ production and activity. Increased ADAMTSL2 levels inhibited myofibroblast differentiation, and attenuated important pro-fibrotic, phenotypic properties of CFBs i.e. proliferation, migration and contractility. Taken together, our data indicate that ADAMTSL2 is a negative regulator of TGFβ and the fibrotic response in CFBs.

A central finding in our study is that increased levels of extracellular ADAMTSL2 attenuated canonical TGFβ signalling in human CFBs, shown through reduced intracellular SMAD activation and nucleus translocation, and reduced expression of specific TGFβ target genes. This is in line with the increased TGFβ levels and activity observed in isolated dermal fibroblasts from patients with GD, caused by *ADAMTSL2* loss-of-function mutations^22^, in HEK293 cells transfected with the GD-causing ADAMTSL2 mutation p.Gly296Arg^33^, and in chondrocytes isolated from *Adamtsl2*−/− mice^34^. Thus, our findings were consistent with the existing literature on non-cardiac cells, and we identified that increased ADAMTSL2 levels resulted in both reduced TGFβ amounts and activity in fibroblasts of the heart. As TGFβ signalling is central to cardiac fibrosis, this is a novel finding with possible implications for limiting fibrosis in heart failure.

TGFβ regulation is extremely complex, with positive and negative feedback loops, and its activity is largely regulated by extracellular activation^35,36^. We found that extracellular ADAMTSL2 did not inhibit the active, free form of TGFβ in the extracellular environment, indicating that the effect of ADAMTSL2 on TGFβ1 must be up-stream, or at the level of, TGFβ1 activation in the ECM. Previous studies have demonstrated that ADAMTSL2 binds directly to LTBP1 and fibrillin-1 and -2^22,24,37,38^, potentially forming a complex in the ECM. Thus, we speculate that ADAMTSL2 might inhibit TGFβ through this interaction. Mechanistically, we suggest that increased extracellular ADAMTSL2 inhibits TGFβ complex deposition in the ECM, through direct binding of the LLC, or through competitive binding of fibrillin microfibrils, thus limiting the amount of TGFβ that is available for activation. Alternatively, ADAMTSL2 may stabilize the LLC on fibrillin microfibrils, preventing TGFβ release, however, this seems less likely as we found reduced levels of LLC in the ECM of L2 cells.

As TGFβ positively regulates its own gene expression^35,39,40^, less TGFβ activity may cause the observed reduction in *TGFB1* expression and protein production in L2 cells. Treatment with active, recombinant TGFβ increased endogenous ADAMTSL2 expression in CFBs, indicating that ADAMTSL2 is part of a negative feedback loop, in which TGFβ increases its own inhibitor. Indeed, ADAMTSL2 expression was recently shown to be controlled by TGFβ during fibrous tissue differentiation in the sclerotome^41^, supporting this theory. However, the molecular mechanisms underlying the reduction in TGFβ production and activity by ADAMTSL2 remain to be elucidated.

Our analysis of multiple facets of CFB phenotype showed that ADAMTSL2 inhibited cardiac myofibroblast differentiation. This was seen through reduced α-SMA and osteopontin expression, signature molecules of the myofibroblast. Additionally, CFB characteristics shifted towards a less fibrotic phenotype, with reduced stress fibre formation, focal adhesion and migration, as well as reduced proliferation, in response to increased ADAMTSL2. The latter is in line with increased proliferation of *Adamtsl2*-deficient chondrocytes^34^. Importantly, the ultimate defining feature of the myofibroblast, contractility^42^, was inhibited by ADAMTSL2. Since expression of α-SMA and osteopontin is essential for development of cardiac fibrosis^29^, our results suggest that ADAMTSL2 reduces pro-fibrotic effects of CFBs. As addition of TGFβ rescued the expression of α-SMA, the effects of ADAMTSL2 on myofibroblast differentiation was likely mediated through TGFβ.

ADAMTSL2 mutations cause recessive GD (MIM #231,050) in humans, a severe connective tissue disorder with poor prognosis, in which cardiac anomalies, such as progressive valve thickening, are found^20^. In beagles, an *Adamtsl2* loss-of-function founder mutation causes Musladin-Lueke Syndrome, with severe skin and intermuscular fibrosis^23^ and *Adamtsl2*-/- mice die shortly after birth due to bronchial occlusion with accumulated fibrillin microfibrils, in addition to cardiac malformations^24^. The phenotypic evidence from ADAMTSL2 mutations in humans, mice and dogs suggests that ADAMTSL2 could limit cardiac fibrosis, possibly through regulation of collagen and microfibrils^24,34,37^. We did not observe changes in collagen synthesis in our CFB cultures, but we found that ADAMTSL2 affected expression of core microfibril components: fibrillin-1, fibrillin-2 and tropoelastin, suggesting that ADAMTSL2 is modulating cardiac ECM composition. However, elastic fibres are difficult to study in culture, and in vivo studies would have to be performed to settle the role of ADAMTSL2 in regulation of microfibrils in the heart.

In the failing heart, adaptive brakes are expressed to counteract the maladaptive drivers. A prime example of an adaptive brake is the hallmark heart failure blood and tissue biomarker brain natriuretic peptide (BNP), which was recently included in the treatment guidelines^1,43^. We speculate that elevated levels of ADAMTSL2 in patients and mice with fibrosis and heart failure could be beneficial, similar to BNP. The lack of experiments examining the in vivo role of ADAMTSL2 in cardiac remodelling, fibrosis and failure is therefore a significant limitation of our study. Such experiments are complicated by the fact that *Adamtsl2*−*/*− mice are non-viable^24^. Nevertheless, heterozygous *Adamtsl2*−*/*+ mice, or mice with global post-natal or fibroblast-specific conditional *Adamtsl2* inactivation could be utilized. Efforts to increase ADAMTSL2 experimentally during heart failure progression, e.g. with an AAV vector, should also be made to evaluate the effects of ADAMTSL2 in vivo. Thus, whether increased cardiac ADAMTSL2 has beneficial effects in the diseased heart remains to be elucidated in future experiments.

In conclusion, the extracellular matrix-localized glycoprotein ADAMTSL2 was upregulated in fibrotic and failing hearts of patients and mice. ADAMTSL2 inhibited TGFβ and myofibroblast differentiation, reduced the expression of pro-fibrotic signature molecules and attenuated central pro-fibrotic properties of human CFBs. Mechanistically, we showed that ADAMTSL2 over-expressing cells produce less TGFβ, and speculate that ADAMTSL2 inhibits TGFβ deposition and activation, contributing to a negative TGFβ feedback loop. We suggest that ADAMTSL2 may have anti-fibrotic effects in the failing heart.

## Methods

An expanded Supplementary Methods section is available in the Supplementary Information.

### Ethics

The human cardiac biopsy protocol was approved by the Regional Committee for Medical Research Ethics (REK IDs 07482a, S-02292/2017–570 and 2010/2226), the South-Eastern Regional Health Authority of Norway, and was in accordance with the Declaration of Helsinki. Informed consent was signed by all patients and the next of kin of heart donors. Mouse protocols were approved by the Norwegian National Animal Research Committee (approval 8041), and conformed to the NIH Guide for the Care and Use of Laboratory Animals (NIH publication no. 85-23, revised 2011) and the ARRIVE guidelines for reporting of animal research^44^.

### Human heart tissue samples

Left ventricular (LV) tissue biopsies were obtained at Oslo University Hospital, Norway, from patients with heart disease of three aetiologies, Aortic stenosis (AS, n = 11), hypertrophic obstructive cardiomyopathy (HOCM, n = 15) and dilated cardiomyopathy (DCM, n = 20). All patients received standard clinical evaluation, treatment and follow-up in accordance with Oslo University Hospital guidelines. Patient characteristics have been previously described^30–32^. For detailed method description, see Supplementary Methods 3.1.

### Mouse pressure overload heart failure model

Mouse heart samples used for this study were derived from a previously published cohort^25^. In brief, experimental heart failure was induced in 8–10 week-old C57BL/6 J mice by aortic banding (AB) of the ascending aorta, causing pressure overload of the LV. Hearts were harvested at two, four and 20 weeks post-AB. Analgesia was administered pre- and post-operatively by subcutaneous injection of (0.3 mg/mL) buprenorphine, with additional analgesics given based on the status of the animal. The mice were euthanized by dissection of the heart under deep terminal anaesthesia breathing 3% isoflurane. For detailed method description, see Supplementary Methods 3.2.

### In-situ hybridization of mouse heart sections

In situ hybridization was performed on sectioned, paraffin-embedded, AB- or sham-operated mouse hearts using an RNAscope technology with a probe specific for mouse *Adamtsl2.* Hematoxylin was used as the counterstain. For full method description, see Supplementary Methods 3.3.

### Cultures of neonatal rat cardiac myocytes and fibroblasts

Primary cultures were prepared as described previously^45^. Briefly, CMs and CFBs were isolated from hearts of 1–3 day old neonatal rats (Wistar). Cells were plated at 3.8 × 10^4^ cells/cm^2^cultured in serum-containing Dulbecco’s Modified Eagle medium in a 37 °C, 5% CO_2_ humidified incubator. The purity of the cultures was determined by expression analysis of the CM-specific gene *Tnni3*, the endothelial cell marker *Vwf,* and the fibroblast markers *Col1a2*, *Postn*, and *Acta2* (see Supplementary Fig. S1a-c). For full method description, see Supplementary Methods 3.4.

### Human cardiac fibroblast cultures

Commercially available human foetal (Cell Applications) and adult (PromoCell) cardiac fibroblasts (hfCFBs and haCFBs, respectively) were used for cell culture experiments with the recommended culture media. In summary, cells were plated at 20,000 cells/cm^2^ and cultured for four days (generating an immature, developing ECM) or 10,000 cells/cm^2^ and cultured for seven days (generating a mature ECM) before harvest (see Supplementary Fig. S3). Cells were kept at 37˚C, in a 5% CO_2_ humidified incubator. Adenoviral transduction for over-expression of *ADAMTSL2* (L2) or vehicle control (Veh) was performed one or four days after seeding in cultures with immature or mature ECM, respectively. Transduction was performed in serum-containing medium for 24 h, followed by serum-free medium for 48 h. Non-transduced cells were treated with conditioned medium, containing ADAMTSL2 protein (L2-medium), or control (Veh-medium) diluted 1:1 in serum-free medium, for three days. Cells were harvested 72 h after seeding. Transduced cells were treated with recombinant TGFβ (10 µg/µL) for 24 h following 24 h serum starvation. Non-transduced cells were treated with recombinant TGFβ (10 µg/µL) and/or TGFβ-SMAD inhibitor (10 µM) for 2 h following 24 h serum starvation. Non-transduced cells were treated with L2 or Veh conditioned medium, pre-incubated for 1 h with recombinant TGFβ1 (10 µg/µL), for 0, 30 and 60 min before harvest. For detailed method description, see Supplementary Methods 3.5.

### Immunocytochemistry

hfCFBs were cultured on coverslips, fixed in 4% PFA, permeabilized and incubated with primary antibodies for α-SMA, fibrillin-1, EDA-fibronectin, LTBP1 and Collagen type I, and fluorescently labelled secondary antibodies. Cells were mounted onto slides and proteins were visualized with the Axioscan Z1 (Carl Zeiss) for full slide scanning, or the LSM 710 confocal microscope (Zeiss). For full method description, see Supplementary Methods 3.6.

### Collagen gel contraction assay

hfCFBs transduced with Veh or L2 were mixed with collagen and added to BSA-coated plates. The gels were allowed to polymerize before serum-free medium was added to release the gels from the surface. Contraction was observed over the next 24 h, percent contraction was calculated by measuring the circumference of the collagen gels. For full method description, see Supplementary Methods 3.7.

### EdU incorporation assay

hfCFBs transduced with Veh or L2 were seeded in serum-containing medium at 10^3^ cells/cm^2^ in 96-well plates. After 24 h, serum-free medium, or serum-containing medium for positive control, was added. After 48 h, cells were labelled with EdU (10 µM) for 2 h. Cells were fixed according to protocol (Click-iT™ EdU Proliferation Assay, Cat# C10499, CyQUANT, Invitrogen) and fluorescence was measured on the Hidex microplate reader.

### Cell monolayer scratch migration assay

hfCFBs were seeded and transduced in 12-well plates, and kept in serum-free medium for 24 h. A 1 mm wide scratch was made through the cell monolayer using a 200 µL pipette tip and fresh serum-free medium was added. Cell migration across the gap was observed and images were taken with the Eclipse Ts100 phase contrast microscope. Percent migration was calculated from the size of the scratch at 24 h using ImageJ (NIH).

### [^3^H] proline incorporation assay

hfCFBs were seeded and transduced in 12-well plates. After 24 h, serum-free medium containing ascorbic acid (50 µM/mL) and L-[2,3-^3^H]-Proline (1 µCi, Cat# NET323001MC, Perkin Elmer) was added. After 48 h, cells were lysed in NaOH (1 M) and diluted in OptiPhase HiSafe 3 liquid scintillation cocktail (Cat# 1200.437, Perkin Elmer). Amount of incorporated radiolabelled proline, a surrogate for collagen biosynthesis^46^, was measured on the Wallac Winspectral 1414 liquid scintillation counter (Perkin Elmer).

### Gene expression analysis

Total RNA was isolated from LVs and cell cultures, and relative gene expression was determined using TaqMan probes (Supplementary Table S1) or TaqMan array plates. Gene expression was normalised to housekeeping genes 60S ribosomal protein L32 (*RPL32*), L4 (*RPL4*), or glyceraldehyde 3-phosphate dehydrogenase (*GAPDH*). For full method description, see Supplementary Methods 3.8.

### Gene expression data mined from available online databases

The Single Cell Expression Atlas database (https://www.ebi.ac.uk/gxa/sc/home, EMBL-EBI, Cambridgeshire, UK, accessed on 11.07.2021) was used to mine for published single cell RNA sequencing data describing *Adamtsl2* expression in different organs and cell types. The Genotype-Tissue Expression (GTEx) Project Portal (https://gtexportal.org/home/, Broad Institute, Boston, MA, accessed on 11.07.2021) was used to analyse ADAMTSL family expression (RNA sequencing) in cultured dermal fibroblasts, atrial appendage and left ventricle of deceased organ donors. Charts were created using the interactive graphics functionality of the databases.

### Protein isolation and immunoblotting

Protein lysates from mouse and human LVs were extracted as previously described^25,45^, using a PBS‐based lysis buffer containing 1% Triton X‐100. Protein lysates from cell cultures were extracted using the buffer above, or a buffer containing 1% SDS for retrieval of ECM proteins. For studying LTBP1 in cytosolic and ECM protein fractions, cells were lysed using a NP-40-based lysis buffer and fractioned through centrifugation. For studying nuclear translocation of pSMAD, cells were lysed and proteins fractioned using the compartment protein extraction kit (Merck Millipore) according to the manufacturer’s protocol. Secreted proteins were harvested from the cell culture medium. Supernatants and lysates were stored at − 20 °C. N-linked oligosaccharides were enzymatically removed from glycosylated proteins using PNGaseF. Western blotting was performed using the Trans-Blot Turbo blotting system (Bio-Rad). Membranes were blocked in 5% non-fat dry milk, casein or BSA, before incubation with primary antibodies (see Supplementary Table S2) and species-specific horseradish peroxidase secondary antibodies. For full method description, see Supplementary Methods 3.9.

### Statistical analyses

Data are expressed as the minimum value, group mean, and maximum value, with all data points shown in graphs, relative to respective controls and to a reference gene/protein. Normal data distribution was evaluated using the Shapiro–Wilk test. Statistical differences were tested in GraphPad Prism 8, using the unpaired Student *t*-test when comparing two groups, the one-way ANOVA with Tukey’s multiple comparisons test when comparing multiple groups, and the two-way repeated measures ANOVA with the Geisser-Greenhouse correction when comparing multiple time-points. *P*-values < 0.05 were considered statistically significant, and exact p-values are given in the figures, unless *P* < 0.001.

## Supplementary Information

Supplementary Information.

## Data Availability

All data generated or analysed during this study are included in this published article and its Supplementary Information.

## Abbreviations

α-SMA: Alpha smooth muscle actin
AB: Aortic banding
ADAMTSL: A disintegrin-like and metalloproteinase domain with thrombospondin type 1 motifs-like
AS: Aortic stenosis
CFB: Cardiac fibroblast
CM: Cardiomyocyte
DCM: Dilated cardiomyopathy
ECM: Extracellular matrix
GD: Geleophysic dysplasia
haCFB: Human adult cardiac fibroblast
hfCFB: Human foetal cardiac fibroblast
HOCM: Hypertrophic obstructive cardiomyopathy
LA: Left atrium
LLC: Large latent complex
LTBP1: Latent TGFβ binding protein
LV: Left ventricle
SLC: Small latent complex
TGFβ: Transforming growth factor beta
