# Supplementary material for: The extracellular matrix glycoprotein ADAMTSL2 is increased in heart failure and inhibits TGFβ signalling in cardiac fibroblasts
Source: Sci Rep. 2021 Oct 5;11:19757. doi: 10.1038/s41598-021-99032-2 (PMC8492753; doi:10.1038/s41598-021-99032-2)
Supplement: Supplementary file 1 — Supplementary Information. [file 41598_2021_99032_MOESM1_ESM.pdf]

Supplementary Information

The extracellular matrix glycoprotein ADAMTSL2 is increased in heart failure and inhibits TGFβ signalling in cardiac fibroblasts

Karoline B. Rypdal[1,2], Pugazendhi M. Erusappan[1,2], A. Olav Melleby[1,2,3], Deborah E. Seifert[4], Sheryl Palmero[1,2], Mari E. Strand[1,2], Theis Tønnessen[1,2,5], Christen P. Dahl[6], Vibeke Almaas[6], Dirk Hubmacher[7], Suneel S. Apte[4], Geir Christensen[1,2], Ida G. Lunde[1,2,*]

[1]Institute for Experimental Medical Research, Oslo University Hospital and University of Oslo, Oslo, Norway. [2]KG Jebsen Cardiac Research Center and Center for Heart Failure Research, University of Oslo, Oslo, Norway. [3]Section of Physiology, Department of Molecular Medicine, Institute for Basic Medical Sciences, University of Oslo, Oslo, Norway. [4]Department of Biomedical Engineering, Cleveland Clinic Lerner Institute, Cleveland, OH, USA. [5]Department of Cardiothoracic Surgery, Oslo University Hospital Ullevaal, Oslo, Norway. [6]Department of Cardiology, Oslo University Hospital Rikshospitalet, Oslo, Norway. [7]Orthopaedic Research Laboratories, Leni & Peter W. May Department of Orthopaedics, Icahn School of Medicine at Mount Sinai, New York, NY, USA.

1. Supplementary Figures

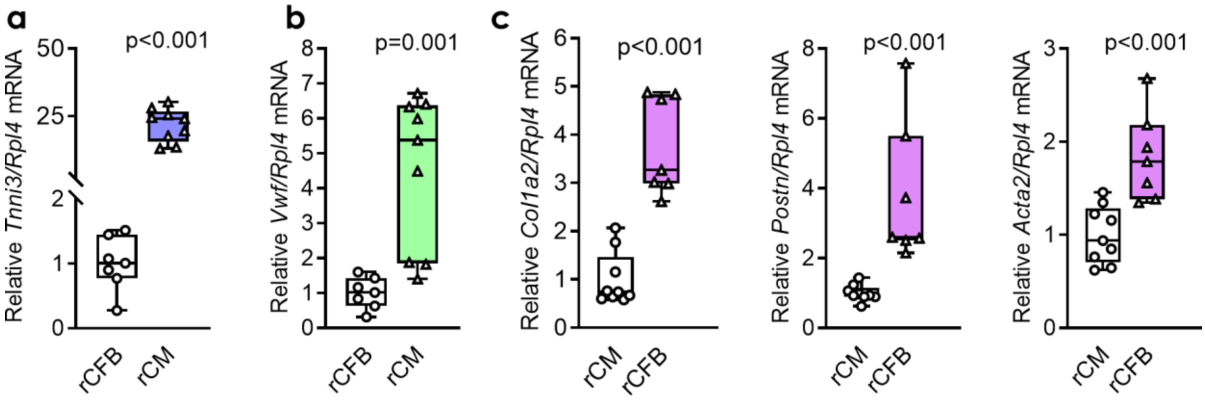

Fig. S1 Purity of primary heart cell cultures prepared from neonatal rats.
Primary heart cell cultures from neonatal rats were prepared to separate cardiomyocytes (rCM) and cardiac fibroblasts (rCFB) according to a standard protocol[1]. **(a)** The purity of the cultures was confirmed by gene expression analysis of the cardiomyocyte-specific marker troponin-I (*Tnni3*). **(b)** Presence of endothelial cells was determined by expression of von willebrand factor (*Vwf*). **(c)** Fibroblast cultures were confirmed by expression of type I collagen (*Col1a2*), periostin (*Postn*), and α-smooth muscle actin (*Acta2*) (myofibroblasts). Gene expression was normalized to *Rpl4.* Data represent n=3 isolations with n=1-3 technical replicates from each isolation. Data are presented as minimum, mean and maximum values, and statistical analysis was performed using the Student *t*-test.

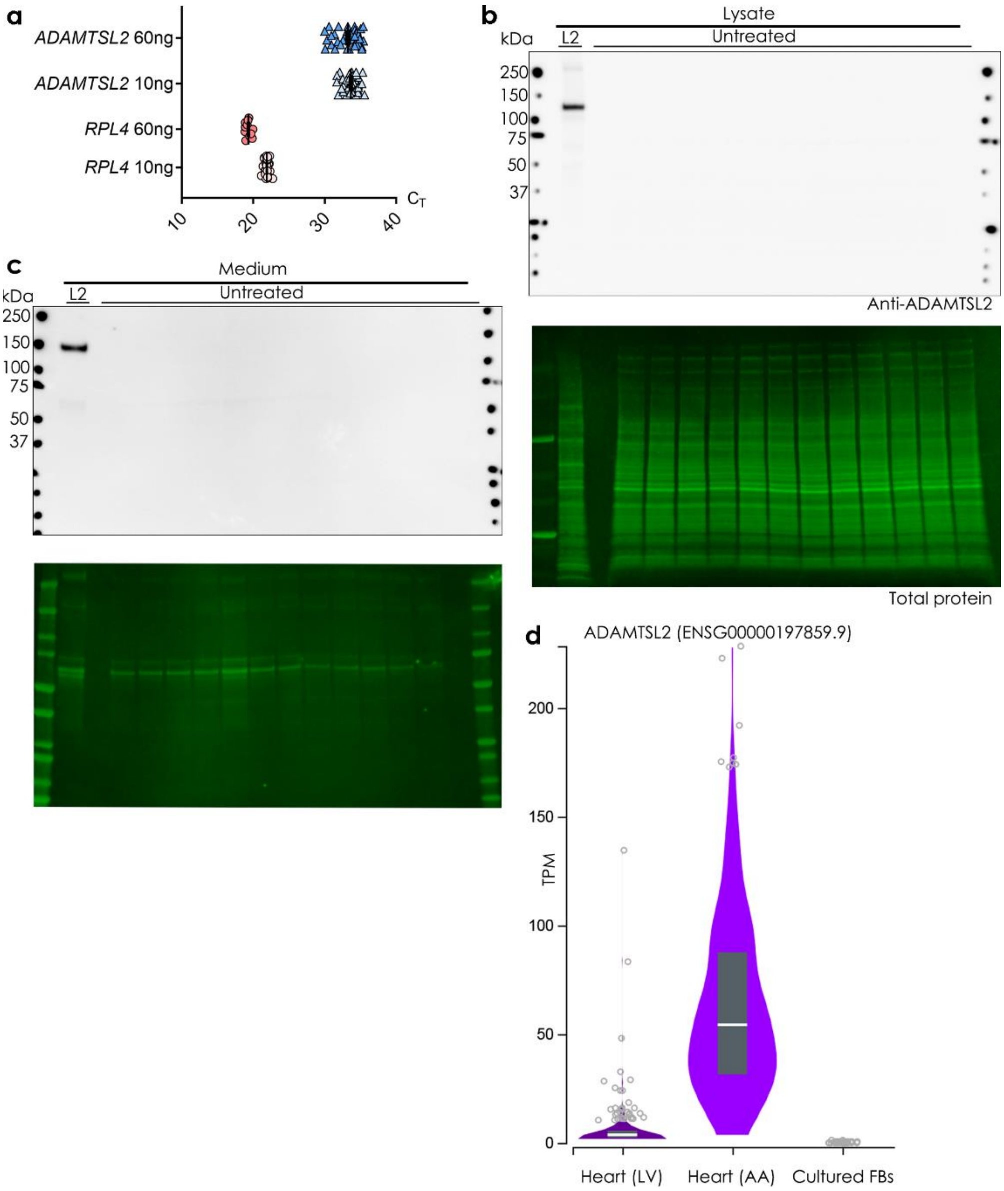

Fig. S2 Negligible ADAMTSL2 expression in cultured human fibroblasts favoured over-expression over siRNA studies to understand the role of ADAMTSL2 in these cells. Human foetal cardiac fibroblasts (hfCFBs) were cultured for two days in growth medium and serum starved for one day before harvest. Data represent experiments from ≥3 different cell passages. **(a)** C_T values from qPCR of *ADAMTSL2* mRNA with 10 ng and 60 ng input, with *RPL4* expression as reference. The *ADAMTSL2* signal is detected at C_T values >30 even with maximum input, in line with negligible expression in untreated hfCFBs. **(b)** Immunoblot of ADAMTSL2 protein in cell lysates (16 μg loaded) and **(c)** cell medium (maximum loading 68 μg), n=9, showing that ADAMTSL2 protein was not detected in, or secreted from, hfCFBs. ADAMTSL2 over-expressing cells (L2) were used as positive control for ADAMTSL2 immunoblot signal. **(d)** RNA sequencing data from the Genotype-Tissue Expression (GTEx) project portal. In GTEx, a total of 17382 samples are collected from 54 different non-diseased tissues considered normal relative to age, across 948 deceased human organ donors, and thus, a control population. Donor age is 20-70 years, 33% female, 85% White and 13% African American. *ADAMTSL2* expression (dbGaP Acession phs000424.v8.p2) shown as transcripts per kilobase million (TPM), in cardiac tissue from the left ventricle (LV, n=432, median TPM=3.55), atrial appendage (AA, n=429, median TPM=54.51) and cultured dermal fibroblasts (FBs, n=504, median TPM=0.05).

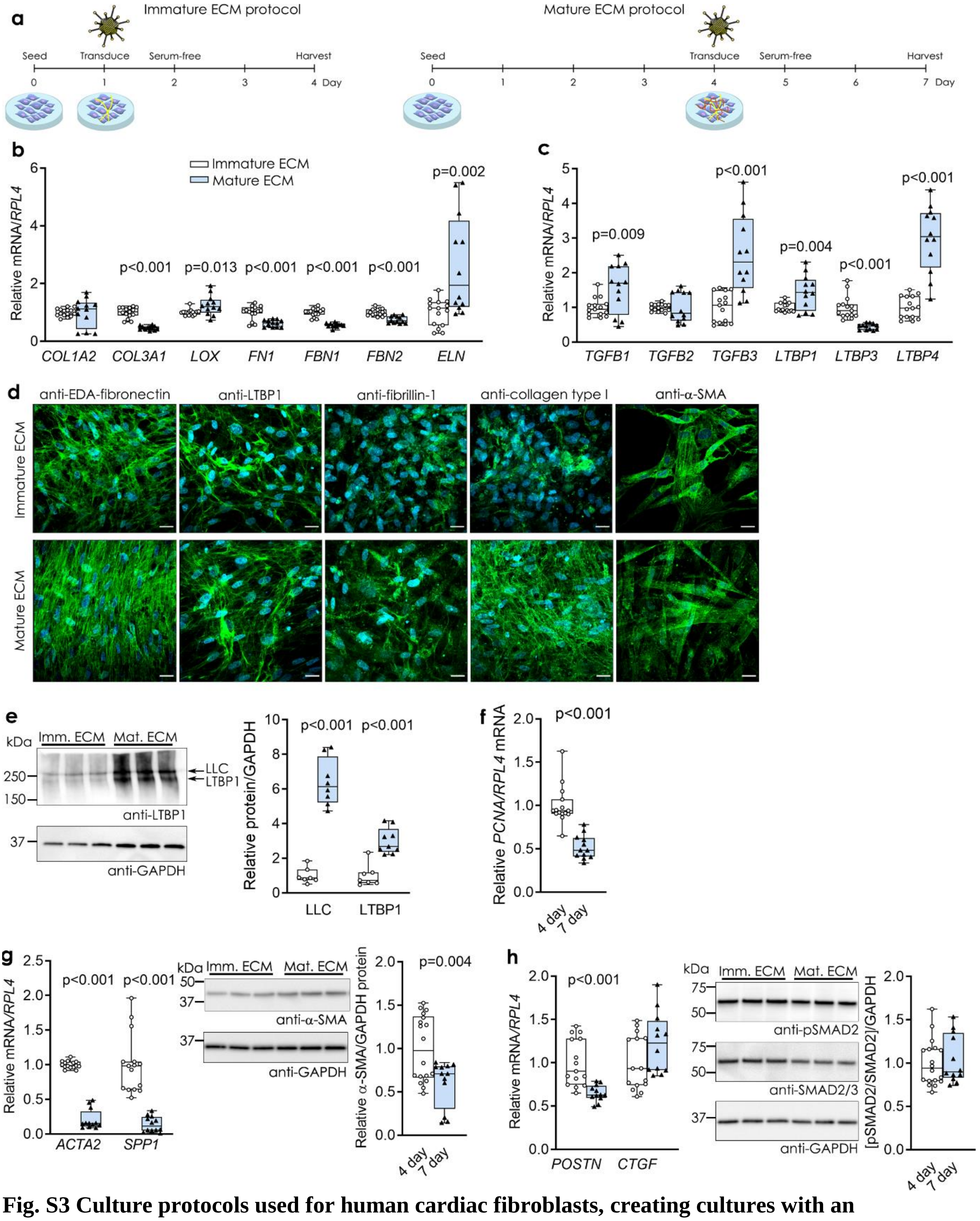

Fig. S3 Culture protocols used for human cardiac fibroblasts, creating cultures with an immature or mature extracellular matrix.

(a) Schematic overview of the human foetal cardiac fibroblasts culture protocol. Cells were cultured for four or seven days, forming an immature or mature extracellular matrix (ECM),

and transduced with ADAMTSL2 or control virus on day one or four, respectively. Data represent experiments from three different cell passages with the respective culture conditions. **(b)** mRNA levels of collagen I (*COL1A2)*, collagen III (*COL3A1*), lysyl oxidase (*LOX*), fibronectin (*FN1*), fibrillin-1, -2 (*FBN1,2*) and elastin (*ELN*) in non-treated mature vs. immature ECM cultures (n=12-15). **(c)** mRNA levels of *TGFB1-3*, *LTBP1, 3 and 4*, in non-treated mature vs immature ECM cultures (n=12-15). **(d)** Representative immunocytochemistry images from three different cell passages of non-transduced mature vs immature ECM cultures, stained for EDA-fibronectin, latent TGFβ binding protein 1 (LTBP1), fibrillin-1, collagen type I and α-smooth muscle actin (α-SMA). Scale bar = 20μM. **(e)** Representative immunoblot of LTBP1 in mature vs immature ECM cultures, showing LTBP1 and the large latent complex (LLC) consisting of LTBP1 and TGFβ, and quantification of intensity from three independent experiments (n=9). **(f)** mRNA levels of proliferating cell nuclear antigen (*PCNA*) in non-treated mature vs immature ECM cultures (n=12-15). **(g)** mRNA levels of α-SMA (encoded by *ACTA2*) and osteopontin (*SPP1*), and representative immunoblots of α-SMA in mature vs immature ECM cultures (n=12-18). **(h)** Representative immunoblots of phosphorylated Smad2 (pSMAD2) and total Smad2/3, and mRNA levels of connective tissue growth factor (*CTGF*) and periostin (*POSTN*), in mature vs immature ECM cultures (n=12-18). All full-size, uncropped blots are available in Supplementary figure VI. Gene expression was normalized to *RPL4*. GAPDH was used for protein loading control. Data are presented as mean ± min/max. Statistical differences were tested using the Student t-test.

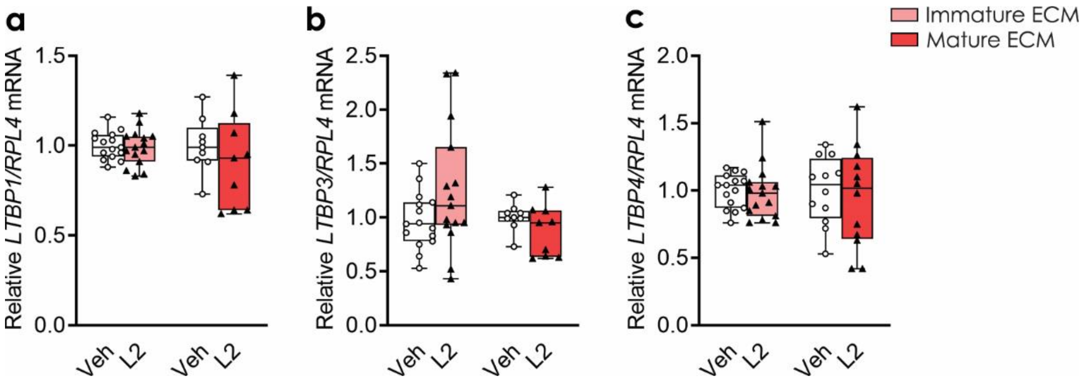

Fig. S4 No difference in LTBP mRNA expression in human cardiac fibroblasts overexpressing ADAMTSL2.

Human foetal cardiac fibroblasts were cultured for four or seven days (see Supplementary Fig. S3a), forming an immature or mature extracellular matrix (ECM), and transduced with ADAMTSL2 (L2) or control (vehicle, Veh) adenoviruses on day one or four, respectively. Data represent experiments from three different cell passages. mRNA levels of **(a)** latent TGFβ-binding protein (*LTBP1)*, **(b)** *LTBP3* and **(c)** *LTBP4* in L2 compared to Veh (n=12-15). Gene expression was normalized *RPL4*. Data are mean ± min/max. Statistical differences were tested using the Student t-test.

Fold change		≤0.33	0.34 - 0.50	0.51-0.67	0.68-0.75	0.68-1.32	1.33-1.49	1.50-1.99	2.0-2.99	≥3.00	Reference	Und
	1	2	3	4	5	6	7	8	9	10	11	12
A	18S	GAPDH	HPRT1	GUSB	ACTB	B2M	RPLP0	HMBS	TBP	PGK1	UBC	PPIA
B	ADAMTS1	ADAMTS13	ADAMTS8	CD44	CDH1	CNTN1	COL11A1	COL12A1	COL14A1	COL15A1	COL16A1	COL1A1
C	COL4A2	COL5A1	COL6A1	COL6A2	COL7A1	COL8A1	VCAN	CTGF	CTNNA1	CTNNB1	CTNND1	CTNND2
D	ECM1	FN1	HAS1	ICAM1	ITGA1	ITGA2	ITGA3	ITGA4	ITGA5	ITGA6	ITGA7	ITGA8
E	ITGAL	ITGAM	ITGAV	ITGB1	ITGB2	ITGB3	ITGB4	ITGB5	KAL1	LAMA1	LAMA2	LAMA3
F	LAMB1	LAMB3	LAMC1	MMP1	MMP10	MMP11	MMP12	MMP13	MMP14	MMP15	MMP16	MMP2
G	MMP3	MMP7	MMP8	MMP9	NCAM1	PECAM1	SELE	SELL	SELP	SGCE	SPARC	SPG7
H	SPP1	TGFBI	THBS1	THBS2	THBS3	TIMP1	TIMP2	TIMP3	CLEC3B	TNC	VCAM1	VTN

Fig. S5 mRNA expression array of extracellular matrix and adhesion proteins in human cardiac fibroblasts overexpressing ADAMTSL2.
Human foetal cardiac fibroblasts were cultured for four or seven days (see Supplementary Fig. S3a), forming an immature or mature extracellular matrix (ECM), and transduced with ADAMTSL2 (L2) or control (vehicle, Veh) adenoviruses on day one or four, respectively. Samples were pooled from three experiments in different cell passages, each with 3-6 technical replicates (n=15). Gene expression analysis was performed using a pre-designed microarray plate containing probes for 84 extracellular matrix and adhesion-related genes and 12 housekeeping genes. The table shows differentially expressed genes in L2 vs. Veh with colour coding according to fold-change (green, upregulated; red, downregulated; white, unchanged). Gene expression was normalized to *GAPDH* using the $2^{-\Delta\Delta CT}$ algorithm.

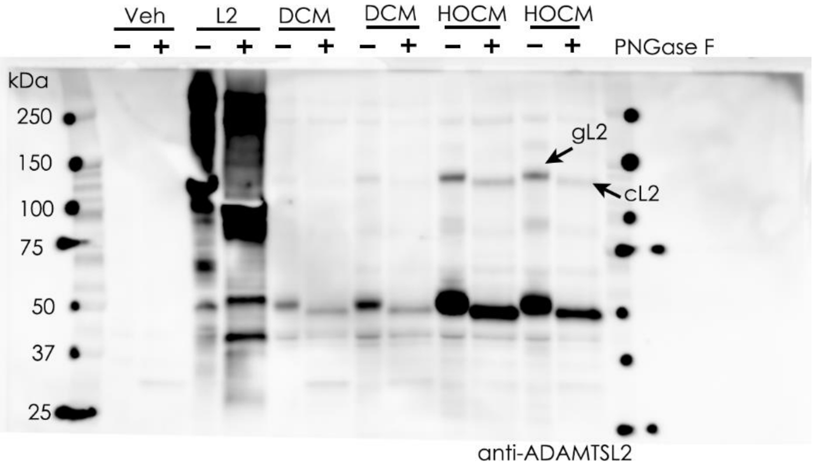

Fig. S6 Immunoblot of ADAMTSL2 with the expected protein bands.
Immunoblot for ADAMTSL2 in left ventricular biopsies from dilated cardiomyopathy (DCM, n=2) and hypertrophic obstructive cardiomyopathy (HOCM, n=2) patients showing a band of the expected size (approx. 140 kDa[2]), representing the full-length, glycosylated ADAMTSL2 protein (gL2). Treatment with PNGase F resulted in de-glycosylated ADAMTSL2 (cL2). Foetal human cardiac fibroblasts over-expressing ADAMTSL2 (L2) or control virus (Veh) were used as positive and negative control, respectively.

2. Supplementary Tables

Table S1 TaqMan gene expression assays used for qPCR.

Gene	*Assay name*	*Species*	*Gene*	*Assay name*	*Species*
ACTA2	Hs00426835_g1	Human	***Adamtsl1***	Mm00553186_m1	Mouse
ADAMTSL2	Hs01115472_m1	Human	***Adamtsl2***	Mm01326794_m1	Mouse
COL1A2	Hs01028956_m1	Human	***Adamtsl3***	Mm01312414_m1	Mouse
COL3A1	Hs00943809_m1	Human	***Adamtsl4***	Mm00523242_m1	Mouse
CTGF	Hs00170014_m1	Human	***Adamtsl5***	Mm01149530_g1	Mouse
ELN	Hs00355783_m1	Human	***Adamtsl6***	Mm01179372_m1	Mouse
FBN1	Hs00171191_m1	Human	***Papln***	Mm01307240_m1	Mouse
FBN2	Hs00266592_m1	Human	***Rpl32***	Mm02528467_g1	Mouse
KI67	Hs00606991_m1	Human	***Rpl4***	Mm01171353_g1	Mouse
LOX	Hs00942482_g1	Human	***Acta2***	Rn01759928_g1	Rat
LTBP1	Hs01558763_m1	Human	***Adamtsl1***	Rn01472249_m1	Rat
LTBP3	Hs01105746_m1	Human	***Adamtsl2***	Rn01439116_m1	Rat
LTBP4	Hs00943217_m1	Human	***Adamtsl3***	Rn01477616_m1	Rat
MCM2	Hs01091564_m1	Human	***Adamtsl4***	Rn01762845_m1	Rat
PCNA	Hs00427214_g1	Human	***Adamtsl5***	Rn01535376_g1	Rat
POSTN	Hs01566750_m1	Human	***Adamtsl6***	Rn00388232_m1	Rat
RPL32	Hs00851655_g1	Human	***Col1a2***	Rn01526720_m1	Rat
RPL4	Hs03044646_g1	Human	***Papln***	Rn01518127_m1	Rat
SPP1	Hs00959010_m1	Human	***Tnni3***	Rn00437164_m1	Rat
TGFB1	Hs00998133_m1	Human	***Vwf***	Rn01492158_m1	Rat
TGFB2	Hs00234244_m1	Human	***Rpl4***	Rn06291926_g1	Rat
TGFB3	Hs01086000_m1	Human			
VCL	Hs00419715_m1	Human			

Table S2 Primary antibodies used for immunoblotting.

Primary antibody	*Vendor, catalogue #*	*Dilution*	*Blocking*	*Condition*
ADAMTSL2	Abcam, #ab97603	1:3000	1% milk	Reducing
Phospho-SMAD2 (Ser465/467)	Cell Signaling, mAb #3108	1:1000	3% BSA	Reducing
SMAD2/3	Cell Signaling, #3102	1:1000	3% BSA	Reducing
LAP (TGFB1)	R&D Systems, #AF-246-NA	1:2000	1% milk	Non-reducing
LTBP1	R&D Systems, #MAB-388	1:1000	1% milk	Non-reducing
α-SMA	Merck, #A5228	1:1000	5% milk	Reducing
Phospho-FAK (Tyr397)	Cell Signaling, #3283	1:1000	5% milk	Reducing
FAK	Cell Signaling, #3285	1:1000	5% milk	Reducing
Phospho-Paxillin (Tyr118)	Cell Signaling, #2541	1:1000	5% milk	Reducing
Paxillin	Cell Signaling, #2542	1:1000	5% milk	Reducing
Vinculin	Merck, #V9131	1:1000	1% milk	Reducing
GAPDH	Santa Cruz Biotechnology, #sc-32233	1:1000	1% milk	Reducing

Table S3. Expression of *ADAMTSL* family genes as median transcripts per kilobase million (TPM) in cardiac tissue and cultured dermal fibroblasts.

Tissue	*Heart (left ventricle)*	*Heart (atrial appendage)*	*Cultured dermal fibroblasts*
Gene	*n=432*	*n=429*	*n=504*
ADAMTSL1	0.17	0.64	31.62
ADAMTSL2	3.55	54.51	0.05
ADAMTSL3	2.54	9.66	0.03
ADAMTSL4	15.63	21.86	21.27
ADAMTSL5	10.66	9.44	6.74
ADAMTSL6	13.11	10.58	7.75
PAPLN	1.07	4.70	3.91

RNA sequencing data from the Genotype-Tissue Expression (GTEx) project portal. In GTEx, a total of 17382 samples are collected from 54 different non-diseased tissues considered normal relative to age, across 948 deceased human organ donors, and thus, a control population. Donor age is 20-70 years, 33% female, 85% White and 13% African American.

3. Supplementary Methods

3.1 Human heart tissue samples

Left ventricular (LV) tissue biopsies were obtained at Oslo University Hospital, from patients with aortic stenosis (AS), hypertrophic obstructive cardiomyopathy (HOCM) and dilated cardiomyopathy (DCM). All patients received standard clinical evaluation, treatment and follow-up in accordance with hospital guidelines.

LV free wall myocardial biopsies (n=11) were taken near the apex of the heart, from AS patients during open-heart surgery for aortic valve replacement. Control LV myocardial biopsies (n=11) were taken from a normally contracting area near the apex of patients undergoing surgery for coronary artery disease. Patient characteristics of this cohort were previously described[3]. In brief, all patients had an ejection fraction (EF) >50%. AS patients had non-dilated LV (LV internal diameter in diastole (LVIDd), 4.81 ± 0.23 cm) and hypertrophic LV walls (interventricular septal thickness in diastole (IVSd), 1.26 ± 0.06 cm and LV posterior wall thickness (LVPWd), 1.22 ± 0.07 cm).

IVS biopsies from HOCM patients (n=15) referred for septal reduction therapy, were obtained during septal myectomy, and patient characteristics were described previously[4]. In brief, the diagnostic criteria were LVPWd >1.5 cm with other causes of hypertrophy excluded, non-dilated LV, and EF>50%.

LV biopsies from DCM patients (n=20) were obtained from beating hearts during heart transplantation. LV tissue from non-diseased hearts considered for transplantation, but deemed unsuitable due to surgical reasons, served as controls for HOCM and DCM (n=3 for RNA samples and n=8 for protein samples). DCM patient and donor characteristics were described previously[5]. In brief, hearts were dilated (LVIDd 7.41 ± 0.22 cm), had reduced systolic function (LVEF of 19.2 ± 1.6%) and walls were not hypertrophic (IVSd 0.81 ± 0.05

cm and LVPWd 0.71 ± 0.02 cm, respectively). Tissue samples were snap-frozen in liquid nitrogen and stored at -70˚C until molecular analysis.

3.2 Mouse pressure overload heart failure model

Mouse heart samples used for this study were derived from a previously published cohort[6]. In brief, experimental heart failure was induced in 8-10 week-old C57BL/6J mice by banding of the ascending aorta using a nitrile O-ring with a fixed inner diameter of 0.61 mm, causing pressure overload of the LV. Phenotypically, the mice developed concentric hypertrophic LV remodelling and reduced LVEF within two weeks, and LV dilatation eight weeks post-aortic banding (AB). At 20 weeks, the mice were in end-stage, dilated heart failure. For surgery, animals were intubated and ventilated, breathing 98% oxygen and 2% isoflurane. Analgesia was administrated pre- and post-operatively by subcutaneous injection of buprenorphine (0.3 mg/mL), with additional analgesics given according to status. The mice were euthanized by dissection of the heart under deep terminal anaesthesia, breathing 3% isoflurane. Hearts were harvested at two weeks (n=7 sham and n=10 AB), four weeks (n=13 sham and n=19 AB) and 20 weeks (n=7 sham and n=10 AB) post-surgery. The LV was excised from beating hearts, rinsed in PBS, snap-frozen in liquid nitrogen and stored at -70°C.

3.3 *In-situ* hybridization of mouse heart sections

AB- or sham-operated 8-10 week-old mouse hearts were fixed in 4% paraformaldehyde (PFA) prior to paraffin embedding. 7 μm thick sections were collected the day before the *in-situ* hybridization experiments and *Adamtsl2* mRNA was detected using the RNAscope technology with a probe specific for mouse *Adamtsl2* (Advanced Cell Diagnostics, Cat. No. 465521). Hybridization was performed in a HybEZ oven and the 2.5 HD Red detection kit was used for visualization. Hematoxylin was used as counterstain. Sections were

photographed using an Olympus BX51 upright microscope (Olympus, Center Valley, PA) with a Leica DFC7000T camera and Leica Application Suite v4.6 imaging software.

3.4 Cultures of neonatal rat cardiac myocytes and fibroblasts

Primary cultures were prepared as previously described[1]. In brief, 1-3 day old, neonatal rats (Wistar, Janvier Labs) were euthanized by decapitation, beating hearts were harvested and the ventricular tissue was digested with collagenase and pancreatin. The non-cardiomyocyte (CM) fraction (i.e. mainly CFBs) was allowed to attach onto uncoated flasks for 20 min in serum-containing Dulbecco's Modified Eagle medium (Gibco), before the non-attached cell fraction (i.e. mainly CMs) was plated onto gelatine and fibronectin-coated plates at 3.8 x 10^4 cells/cm^2. Cells were cultured in 5% CO_2 at 37˚C, in a humidified incubator for one week. CFBs were passaged and seeded for experiments at 3.8 x 10^4 cells/cm^2. The purity of the cultures was determined by expression analysis of the CM-specific gene *Tnni3*, the endothelial cell marker *Vwf*, and the fibroblast markers *Col1a2*, *Postn*, and *Acta2* (see Supplementary Fig. S1a-c).

3.5 Human cardiac fibroblast cultures

Human foetal cardiac fibroblasts (hfCFBs) (Cat# 306-05f, Cell Applications, Inc., San Diego, CA, USA) were cultured in Cardiac Fibroblast Growth medium (Cat# 316-500, Cell Applications) for serum-containing conditions, or Fibroblast Basal medium (Cat# 115-500, Cell Applications) for serum-free conditions. Primary human CFBs isolated from the ventricles of the adult heart (haCFBs) (PromoCell GmbH, Cat# C-12375, Heidelberg, Germany) were cultured in Fibroblast Growth medium 3 (Cat# C-23130, PromoCell) for serum-free conditions or supplemented with 10% Growth Medium 3 Supplement Mix (Cat# C-39345 PromoCell) for serum-containing conditions. All media were supplemented with 1%

penicillin/streptomycin. Cells were cultured at 37˚C, in a 5% CO_2 humidified incubator. Unless otherwise stated, cells were plated at 20,000 cells/cm^2 and cultured for four days (generating an immature, developing ECM) or plated at 10,000 cells/cm^2 and cultured for seven days (generating a mature ECM) before harvesting (see Supplementary Fig. S3a).

hfCFBs and haCFBs were transduced using replication-deficient human adenovirus type 5 (Ad5 dE1/E3) encoding *ADAMTSL2* (Genbank RefSeq BC128389) under the cytomegalovirus (CMV) promoter (L2), or a vehicle control (Ad5 dE1/E3-CMV-Null) (Veh) (Vector Biolabs, Malvern, PA). Transduction was performed at 5 x 10^6 plaque forming units (PFU) with a multiplicity of infection (MOI) of 100, one or four days after seeding, for over-expression in cultures with immature or mature ECM network, respectively (see Supplementary Fig S3a). Transduction was performed in serum-containing medium for 24 h, followed by culture in serum-free medium for 48 h. For experiments with TGFβ stimulation, the cells were treated with recombinant TGFβ1 (10 µg/µL) (Cat# GF111, Merck), 24 h before harvest.

Non-transduced hfCFBs were seeded onto 6-well plates in serum-containing medium. After 24 h, the medium was replaced with conditioned medium containing ADAMTSL2 protein, harvested from hfCFBs transduced with L2 (4-day protocol), or Veh. Conditioned medium was prepared by diluting harvested medium from cells 1:1 with serum-free medium. After another 24 h, the medium was replaced with fresh conditioned medium again, and finally, cells were harvested 72 h after seeding.

Non-transduced hfCFBs were seeded onto 6-well plates in serum-containing medium. After 24 h, the cells were washed with PBS and the medium was replaced with fresh serum-free medium. After another 2 h, the cells were treated with recombinant TGFβ1 (10 µg/µL) (Cat# GF111, Merck) and/or TGFβ-SMAD inhibitor (10 µM) (Cat# SB431542, Selleck Chemicals), for 2 h before harvest.

For experiments with conditioned medium and TGFβ stimulation, non-transduced hfCFBs were seeded onto 6-well plates in serum-containing medium. After 24 h, the cells were washed with PBS and the medium was replaced with fresh serum-free medium. After 3 h, L2 or Veh conditioned medium, pre-incubated for 1 h with recombinant TGFβ1 (10 µg/µL), was added to the cells. Cells were harvested at T0, T30 and T60 minutes after stimulation.

3.6 Immunocytochemistry

hfCFBs were seeded onto coverslips in 24-well plates. Cells were transduced with Veh or L2, or not transduced, and cultured for a total of four or seven days, with serum until the last 48 hours. Cells were washed twice with PBS and fixed in 4% PFA (Cat# 28906, Thermo Fisher Scientific) for 10 min. Fixed cells were washed thrice in PBS and permeabilized in 0.5% Triton X-100 for 10 min. Non-specific binding was blocked with PBS containing 5% BSA and 0.1% Triton X-100 for 10 min. Cells were incubated with primary antibodies anti-αSMA (1:400, Cat# A5228, Sigma), anti-EDA FN1 (1:400, Cat# F1640, Sigma), anti-LTBP1 (1:400, Cat# MAB388, R&D Systems), anti-COL1A1 (1:400, Cat# R1038, ORIGENE), and anti-FBN1 (1:200, Cat# HPA021057, Prestige Antibodies® Powered by Atlas Antibodies) for 1 h at 37°C and washed twice with PBS containing 0.05% Tween-20 (PBS-Tween), followed by a PBS wash. Cells were incubated with fluorescently labelled secondary antibody Alexa Fluor 647 goat anti-mouse (1:500, Cat# A21236, Invitrogen), Alexa Fluor 488 goat anti-rabbit (1:500, Cat# A11034, Invitrogen) and Alexa Fluor 546 phalloidin (1:500, Cat# A22283, Invitrogen) for 1 h, washed thrice with PBS-Tween, once with PBS, and once with H_2O. Cells were mounted with Prolong Diamond Antifade Mountant with DAPI (Cat# P36962, Thermo Fisher Scientific). Proteins were visualized with the Axioscan Z1 (Carl Zeiss, US) for full slide scanning, or the LSM 710 confocal microscope (Zeiss).

3.7 Collagen gel contraction assay

24-well plates were blocked with 2% BSA in PBS at 37°C overnight. hfCFBs transduced with Veh or L2 were trypsinized (Trypsin-EDTA (0.25%), Thermo Fisher Scientific), collected in serum-containing medium (Cell Applications), and centrifuged for 10 min at 120 rcf. Cells were resuspended in serum-free medium (Cell Applications), and centrifugation and resuspension was repeated twice to eliminate residual serum. A collagen solution was prepared by combining five parts of 2x DMEM (SLM-202-B, Merck Millipore), one part of HEPES (0.2M, pH 8.0) and four parts of Collagen I (3 mg/ml, Bovine PureCol®, Advanced BioMatrix). A cell-collagen gel mixture was made to a final concentration of 36.5 x 10^3 cells/cm^2, and added to the BSA-coated plates. The gels were allowed to polymerize for 2 h at 37°C, before serum-free medium was added to release the collagen gel from the plastic surface. Contraction was observed over the next 24 h, and images were taken at six and 24 hours. The circumferences of the collagen gels were measured using ImageJ (NIH), and percent contraction from baseline was calculated.

3.8 Gene expression analysis

Total RNA was isolated from LVs or cell cultures using the RNeasy Mini Kit (Cat# 74106, Qiagen Nordic, Oslo, Norway) according to the manufacturer's protocol. Reverse transcription and cDNA generation was performed using iScript cDNA Synthesis Kit (Cat# 1708891, Bio-Rad Laboratories, Inc., Hercules, CA). Gene expression was determined using TaqMan Gene Expression Assays (Table S1) or the TaqMan gene expression array for human extracellular matrix and adhesion molecules (Cat# 4414133), with TaqMan Universal PCR Master Mix (Cat# 4304437). Detection was performed on the QuantStudio 3 Real-Time PCR System (Applied Biosystems, Foster City, CA). Gene expression was normalized to

expression of 60S ribosomal protein L32 (*RPL32*) or L4 (*RPL4*) housekeeping genes for tissue or cell culture samples, respectively, and glyceraldehyde 3-phosphate dehydrogenase (*GAPDH*) in the gene expression array.

3.9 Protein isolation and immunoblotting

Unless otherwise stated, all reagents were purchased from Merck KGaA. Proteins were extracted from mouse and human LVs as previously described[1,6], using a PBS-based lysis buffer containing 1% Triton X-100, 0.1% Tween-20, protease inhibitors (cOmplete EDTA-free tablets) and PhosStop phosphatase inhibitors (both from Roche Diagnostics).

Whole-cell protein lysates from cell cultures were extracted using the buffer above, or with lysis buffer containing 1% SDS, 31.5 mM Tris-HCl, pH 6.8, and sonicated twice for 8 seconds using a Branson Sonifier® S-150D (Emerson Electric Co. St. Louis, MO).

For investigating LTBP1 and LLC in cytosolic and ECM protein fractions, cells were lysed using a lysis buffer containing 10 mM Tris-HCl, 150 mM NaCl, 0.5% NP-40, 1 mM $MgCl_2$, and 1 mM $CaCl_2$. The lysates were centrifuged at 14.000 rcf for 15 min, with the supernatant containing the cytosolic proteins. The pellet was resuspended in a lysis buffer containing 1% SDS, 31.5 mM Tris-HCl, pH 6.8 to solubilize the ECM, and sonicated twice for 8 seconds. The lysates were centrifuged at 14.000 rcf for 15 min again, with the supernatant containing the ECM fraction. For studying nuclear translocation of pSMAD, cells were lysed and the proteins fractioned using the compartment protein extraction kit (Cat# 2145, Merck Millipore) according to the manufacturer's protocol.

Proteins were quantified using the Micro BCA protein assay kit (Cat# 23235, Thermo Fisher Scientific). N-linked oligosaccharides were removed from glycosylated proteins, including ADAMTSL2, using enzymatic digestion with PNGaseF after protein denaturation, according to protocol (Cat# P0704, New England Biolabs, United Kingdom).

Proteins were separated by SDS-PAGE with pre-cast Criterion TGX gels (Cat# 5671084, Bio-Rad) and semi-dry blotted onto Trans-Blot Turbo polyvinylidene difluoride membranes (Cat# 1704156, Bio-Rad) on the Trans-Blot Turbo blotting instrument (Bio-Rad). Membranes were blocked for 1 h in 5% non-fat dry milk, casein (Roche Diagnostics), or BSA (Bio-Rad), and washed with TBS/Tween 20 prior to incubation with primary antibodies (Table S2) at 4°C overnight. Membranes were incubated with species-specific horseradish peroxidase secondary antibodies for 1 h before detection using ECL Prime Western Blotting System (Cat# RPN2232, GE Healthcare) and the ImageQuant LAS 4000 camera system (GE Healthcare).

4. Supplementary blots

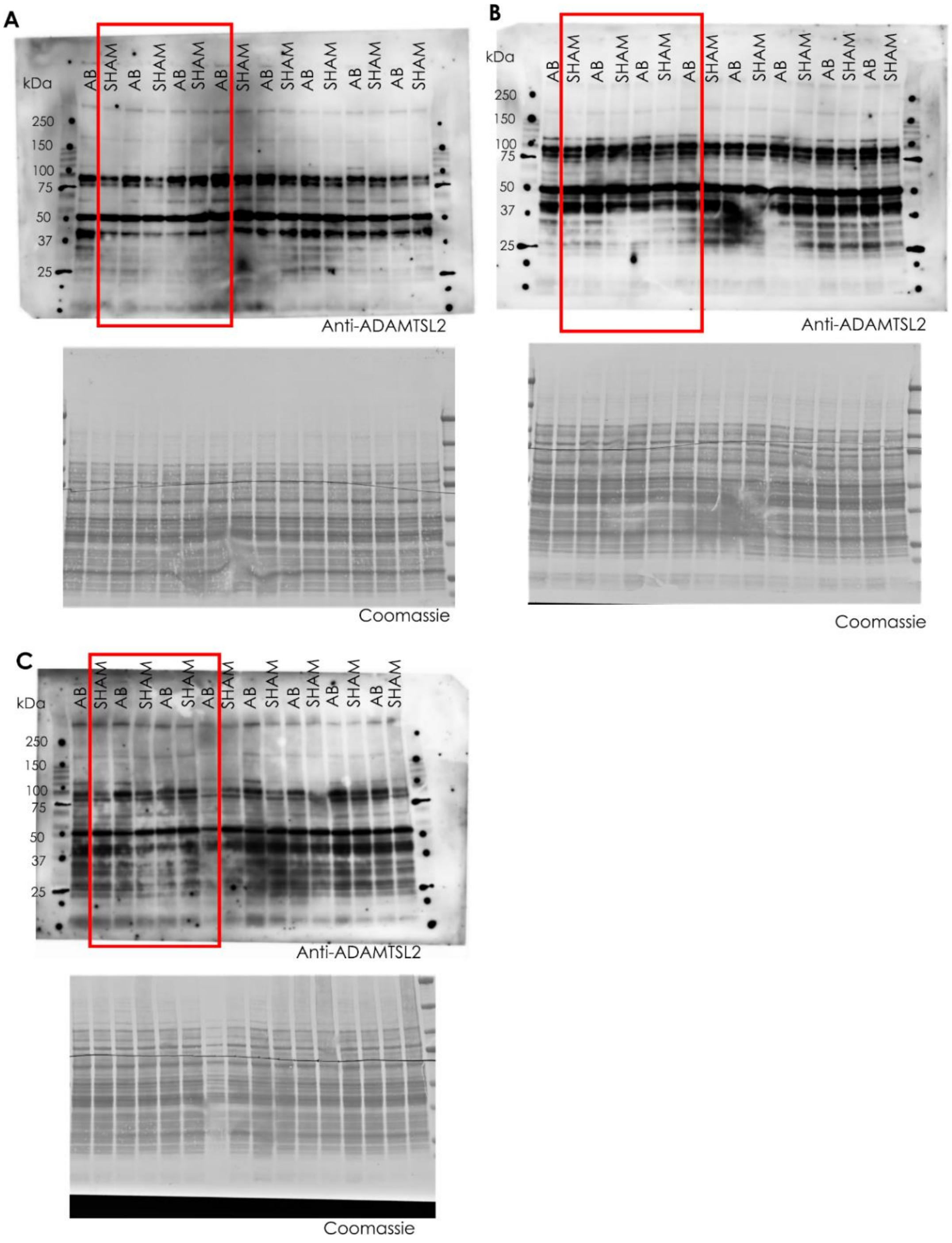

Fig. SI. Full size images of the blot corresponding to Fig. 1b.
Full size blot of left ventricle lysates from aortic banded (AB) or sham-operated (SHAM) mice. Top panels show immunoblot for anti-ADAMTSL2 at 2 weeks (A), 4 weeks (B) and 20 weeks (C) bottom panel shows Coomassie blue staining of the blot. The red boxes indicate the wells that used to make the manuscript figures.

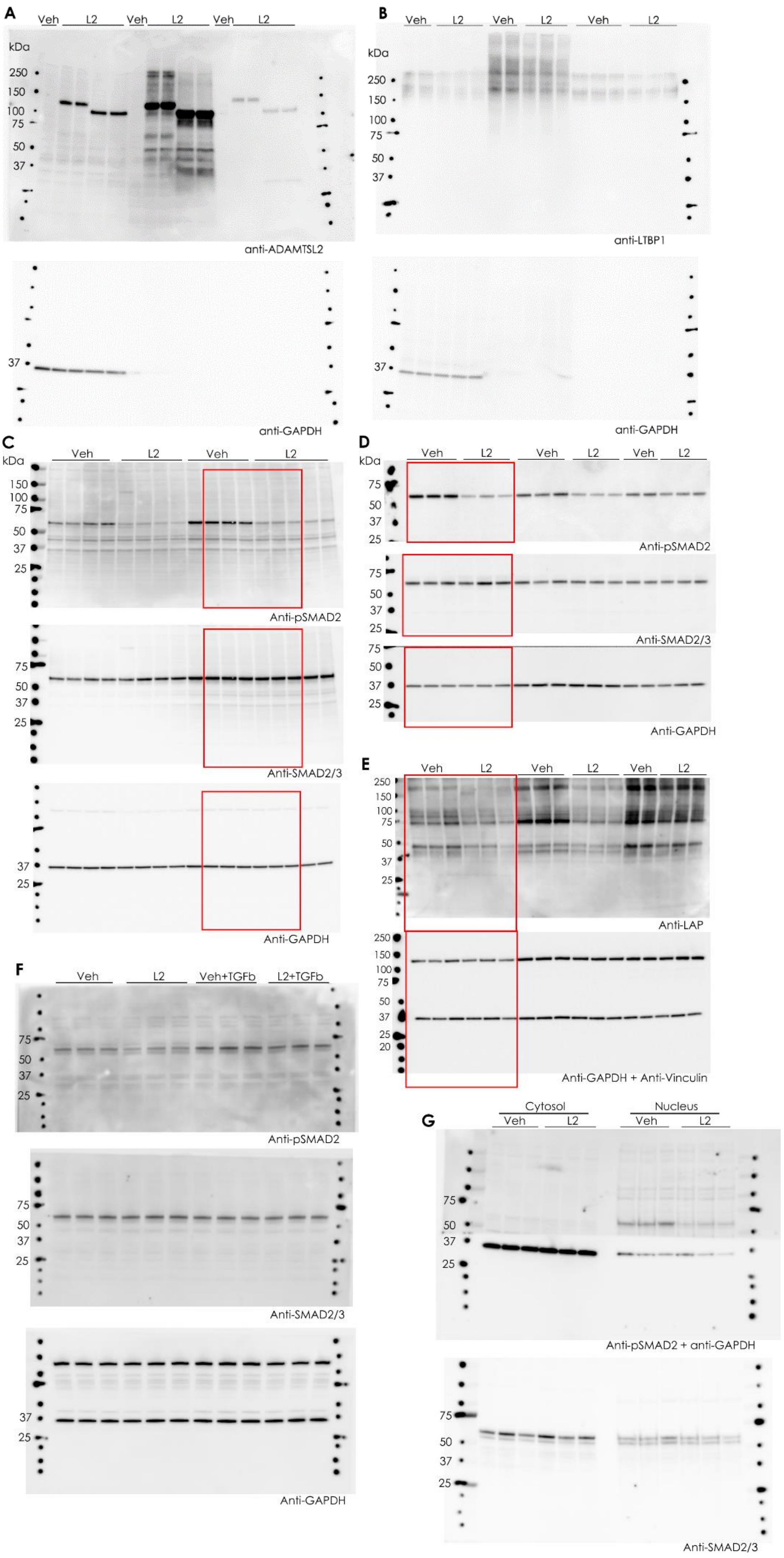
A
Veh
L2
kDa
250
150
100
75
50
37
anti-ADAMTSL2
anti-GAPDH
B
anti-LTBP1
C
Anti-pSMAD2
Anti-SMAD2/3
Anti-GAPDH
D
E
Anti-LAP
Anti-GAPDH + Anti-Vinculin
F
Veh+TGFb
L2+TGFb
G
Cytosol
Nucleus
Anti-pSMAD2 + anti-GAPDH

Fig. SII. Full size images of the blots corresponding to Fig. 2.
All gels are loaded with lysate or medium samples from cultured human foetal cardiac fibroblasts over-expressing ADAMTSL2 (L2) or vehicle control (Veh). Red boxes indicate wells that were used to make the manuscript figure. (A) Full size image of the blot used to make Fig. 2b. Top panel shows immunoblot for anti-ADAMTSL2, bottom panel shows immunoblot for anti-GAPDH as loading control. (B) Full size image of the blot used to make Fig. 2j. Top panel shows immunoblot for anti-LTBP1, bottom panel shows immunoblot for anti-GAPDH as loading control. (C) Full size image of the blot used to make Fig. 2d, with the immature ECM condition. Top panel shows immunoblot for anti-pSMAD2, middle panel shows immunoblot for anti-SMAD2/3, bottom panel shows immunoblot for anti-GAPDH as loading control. (D) Full size image of the blot used to make Fig. 2d, with the mature ECM condition. Top panel shows immunoblot for anti-pSMAD2, middle panel shows immunoblot for anti-SMAD2/3, bottom panel shows immunoblot for anti-GAPDH as loading control. (E) Full size image of the blot used to make Fig. 2g. Top panel shows immunoblot for anti-LAP, bottom panel shows immunoblot for anti-GAPDH as loading control. (F) Full size image of the blot used to make Fig. 2f, with the immature ECM condition. Top panel shows immunoblot for anti-pSMAD2, middle panel shows immunoblot for anti-SMAD2/3, bottom panel shows immunoblot for anti-GAPDH as loading control. All wells were used to make the figure. (G) Full size image of the blot used to make Fig. 2e, with the immature ECM condition. Top panel shows a membrane cut in two, with immunoblot for anti-pSMAD2 (top) and GAPDH (bottom), and the bottom panel shows immunoblot for anti-SMAD2/3. All wells were used to make the figure.

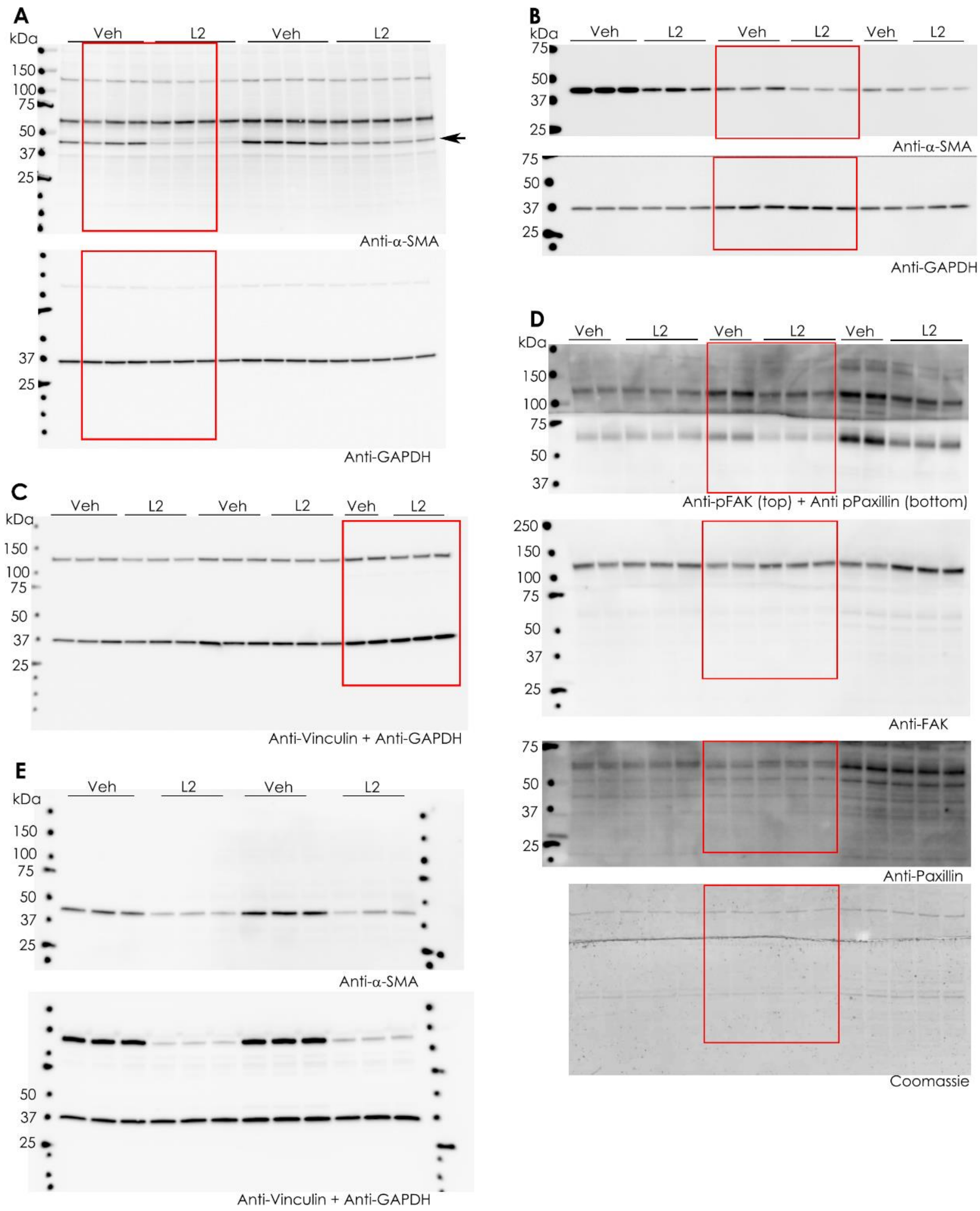

Fig. SIII. Full size images of the blots corresponding to Fig. 4.
All gels are loaded with lysate or medium samples from cultured human foetal cardiac fibroblasts over-expressing ADAMTSL2 (L2) or vehicle control (Veh). Red boxes indicate wells that were used to make the manuscript figure. (A) Full size images of the blot used to make Fig. 4c with the immature ECM condition. Top panel shows immunoblot for anti-α-smooth muscle actin (α-SMA), bottom panel shows immunoblot for anti-GAPDH as loading control. (B) Full size images of the blot used to make Fig. 4c with the mature ECM condition. Top panel shows immunoblot for anti-α-smooth muscle actin (α-SMA), bottom panel shows immunoblot for anti-GAPDH as loading control. (C) Full size images of the blot used to make Fig. 4e with the immature ECM condition. The top part of the gel shows immunoblot for anti-vinculin, and the bottom part shows immunoblot for anti-GAPDH as loading control (mixed antibodies). (D) Full size images of the blot used to make Fig. 4f. The membrane was cut to probe with different antibodies. The top panel shows immunoblot for anti-pFAK (top part of blot) and anti-pPaxillin (lower part of blot). Next, immunoblot for anti-FAK, immunoblot for anti-Paxillin, and the bottom panel shows Coomassie blue staining as loading control. (E) Full size images of the blot used to make Fig. 4l with and without adding recombinant TGFβ in the immature ECM condition. Top panel shows immunoblot for anti-α-smooth muscle actin (α-SMA), bottom panel shows immunoblot for anti-GAPDH (36 kDa) as loading control.

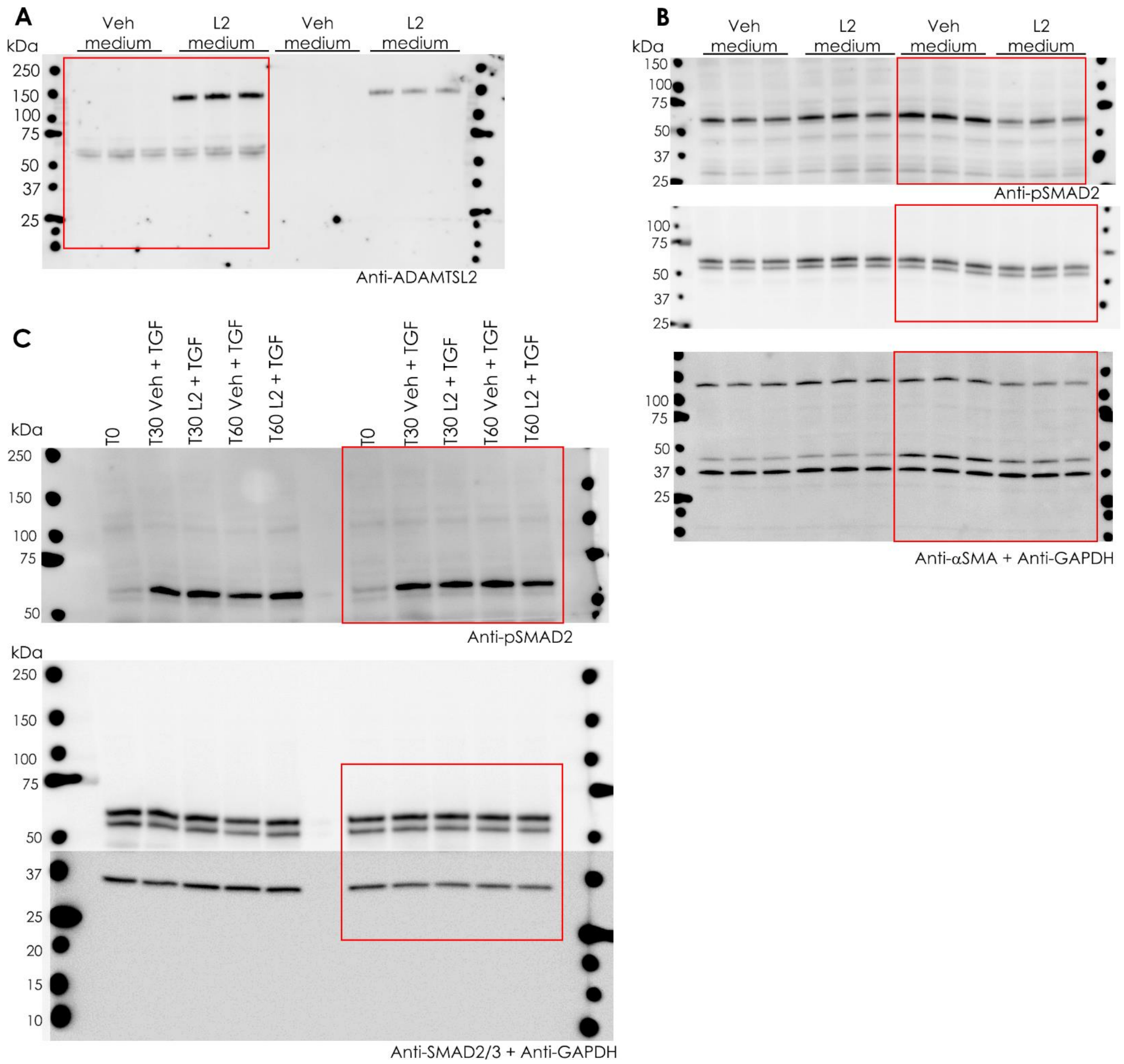

Fig. SIV. Full size images of the blots corresponding to Fig. 5.
(A) Full size image of the blot used to make Fig. 5a, immunoblotting for anti-ADAMTSL2. The gel was loaded with conditioned medium from cultured human foetal cardiac fibroblasts (hfCFBs)over-expressing ADAMTSL2 (L2-medium) or vehicle control (Veh-medium). (B) Full size images of the blot used to make Fig. 5c, immunoblotting for anti-pSMAD2 (top), anti-SMAD2/3 (middle), and anti-α-smooth muscle actin (α-SMA, 42 kDa) and anti-GAPDH (36 kDa) as loading control (bottom). The gel was loaded with lysates from cultured hfCFBs treated with L2-medium or Veh-medium. (C) Full size images of the blot used to make Fig. 5e, immunoblotting for anti-pSMAD2 (top), anti-SMAD2/3 (middle), and anti-GAPDH (bottom) as loading control. The gel was loaded with lysates from cultured hfCFBs treated for 0 (T0), 30 (T30) or 60 (T60) minutes with L2-medium or Veh-medium that was pre-incubated with TGFβ. The membranes were cut to probe for different antibodies. Red boxes indicate the wells that were used to make the manuscript figure.

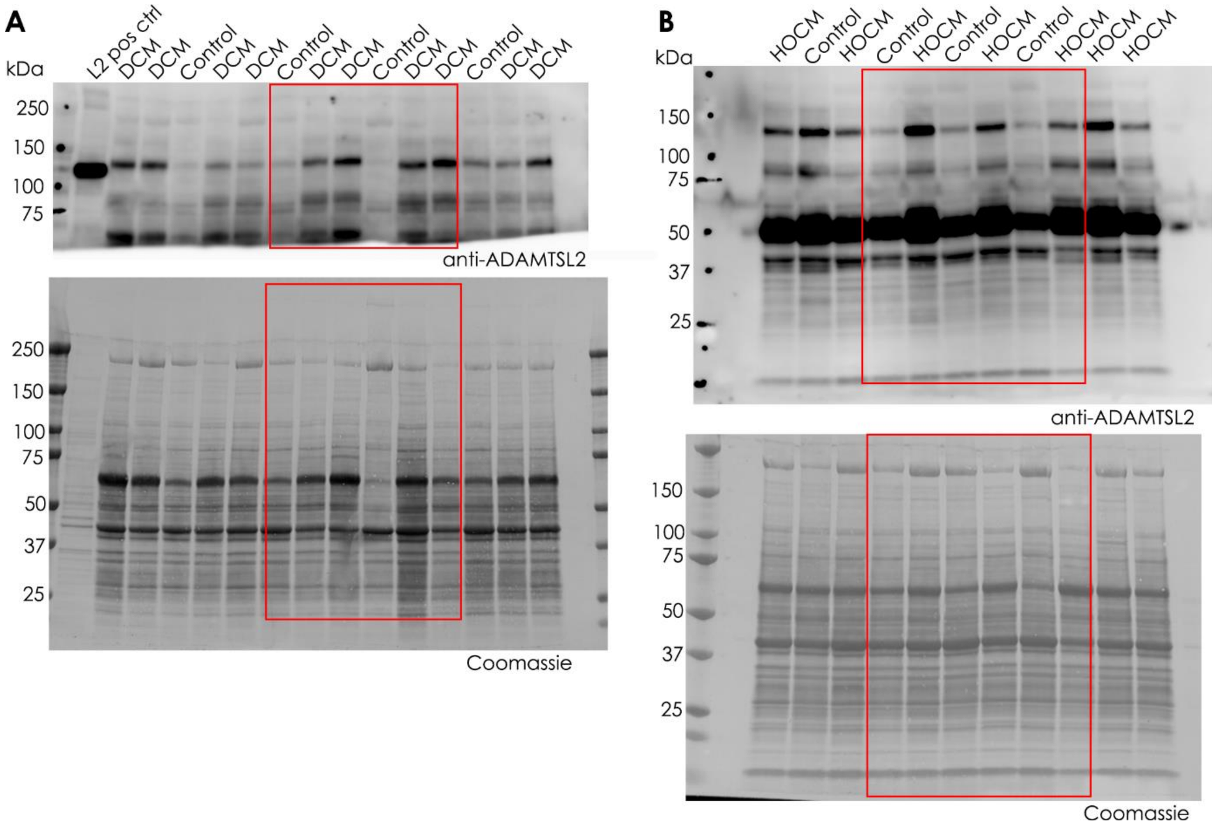

Fig. SV. Full size images of the blots corresponding to Fig. 6.
(A) Full size image of the blot used to make Fig. 6b. The gel was loaded with left ventricle biopsy lysates from patients with dilated cardiomyopathy (DCM) or control donor hearts. Well number one was loaded with a positive control for ADAMTSL2 protein (human foetal cardiac fibroblasts over-expressing ADAMTSL2). The top panel shows immunoblot of anti-ADAMTSL2 (bottom half of gel was covered with paper to avoid over-exposure of lower part) and the bottom panel shows Coomassie blue staining as loading control. (B) Full size image of the blot used to make Fig. 6a. The gel was loaded with myocardial biopsy lysates from patients with hypertrophic obstructive cardiomyopathy (HOCM) or control donor hearts. The top panel shows immunoblot of anti-ADAMTSL2 and the bottom panel shows Coomassie blue staining as loading control. Red boxes indicate the wells that were used to make the manuscript figure.

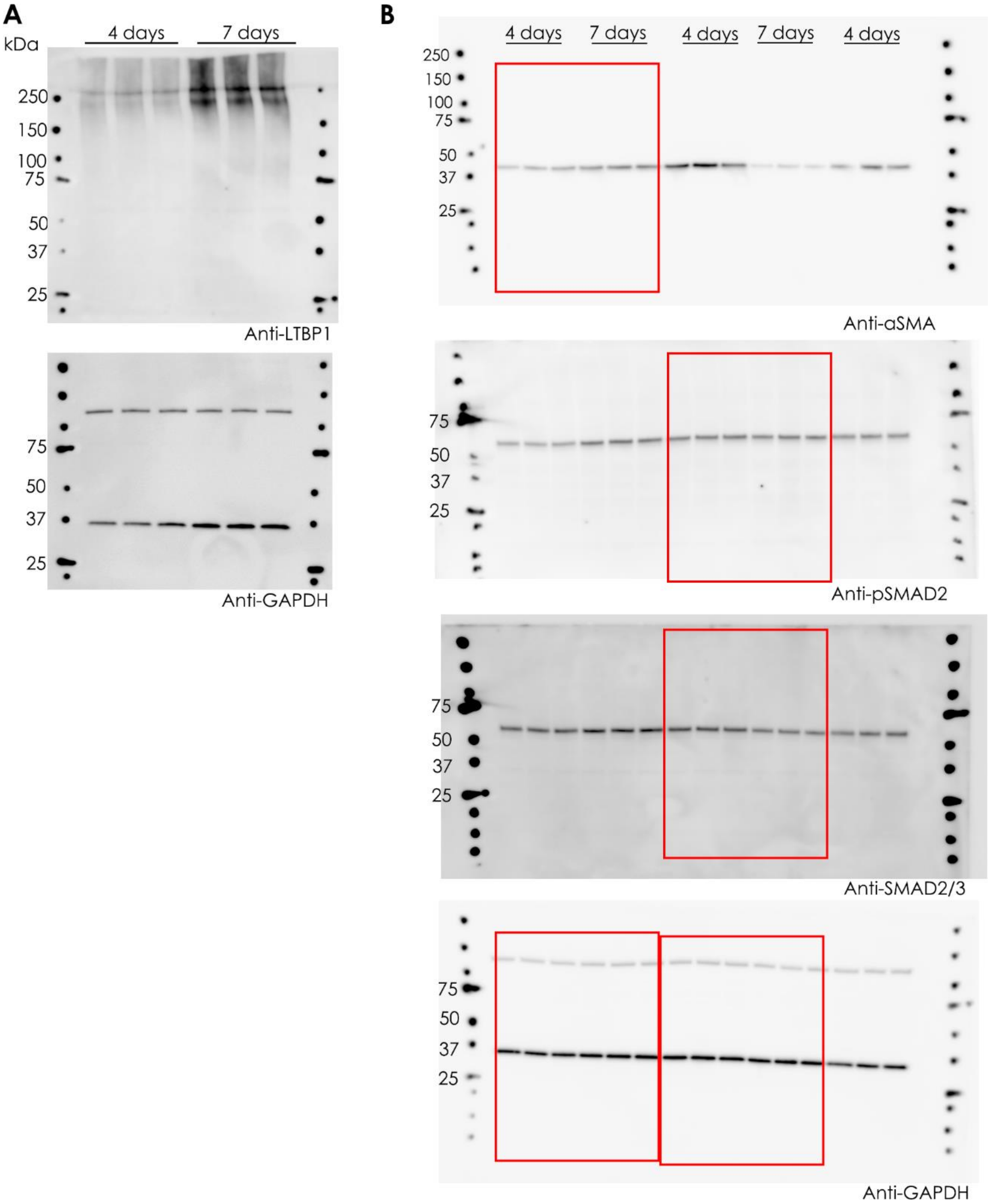

Fig. SVI. Full size images of the blots corresponding to Fig. S3.
(A) Full size image of the blot used to make Fig. S3e, immunoblotting for anti-LTBP1. (B) Full size images of the blot used to make Fig. S3g-h, immunoblotting for anti α-SMA (top), anti-pSMAD2 (second from top), anti-SMAD2/3 (second from bottom), and anti-GAPDH (36 kDa) as loading control (bottom). Both gels were loaded with lysates from cultured human foetal cardiac fibroblasts that were grown for four or seven days in total. Red boxes indicate the wells that were used to make the Supplementary figure.

5 References

1 Strand, M. E. *et al.* Innate immune signaling induces expression and shedding of the heparan sulfate proteoglycan syndecan-4 in cardiac fibroblasts and myocytes, affecting inflammation in the pressure-overloaded heart. *FEBS J* **280**, 2228-2247, (2013).

2 Koo, B. H. *et al.* ADAMTS-like 2 (ADAMTSL2) is a secreted glycoprotein that is widely expressed during mouse embryogenesis and is regulated during skeletal myogenesis. *Matrix Biol.* **26**, 431-441, (2007).

3 Herum, K. M. *et al.* Syndecan-4 protects the heart from the profibrotic effects of thrombin-cleaved osteopontin. *J Am Heart Assoc* **9**, e013518, (2020).

4 Almaas, V. M. *et al.* Increased amount of interstitial fibrosis predicts ventricular arrhythmias, and is associated with reduced myocardial septal function in patients with obstructive hypertrophic cardiomyopathy. *Europace* **15**, 1319-1327, (2013).

5 Melleby, A. O. *et al.* The heparan sulfate proteoglycan glypican-6 is upregulated in the failing heart, and regulates cardiomyocyte growth through ERK1/2 signaling. *PLoS One* **11**, e0165079, (2016).

6 Melleby, A. O. *et al.* A novel method for high precision aortic constriction that allows for generation of specific cardiac phenotypes in mice. *Cardiovasc. Res.* **114**, 1680-1690, (2018).
